# Analyses on patterns of lymph node metastasis and its impact on prognosis in thoracic esophageal squamous cell carcinoma treated with neoadjuvant immunochemotherapy versus chemotherapy alone: a single-center, retrospective cohort study

**DOI:** 10.3389/fimmu.2026.1762746

**Published:** 2026-03-04

**Authors:** Weibin Liu, Yujie Deng, Xuejin Zheng, Weikun Su, Jianqing Zheng, Yijin Lin, Weijin Xiao, Yi Shi, Jiarong Zhang, Weimin Fang, Xiaohui Chen

**Affiliations:** 1 Department of Thoracic Surgery, Clinical Oncology School of Fujian Medical University, Fujian Cancer Hospital, National Health Commission (NHC) Key Laboratory of Cancer Metabolism, Fuzhou, China; 2 Department of Medical Oncology, The First Affiliated Hospital of Fujian Medical University, Fuzhou, Fujian, China; 3 Department of Medical Oncology, Fuzhou Hospital of Traditional Chinese Medicine affiliated to Fujian University of Traditional Chinese Medicine, Fuzhou, Fujian, China; 4 Interdisciplinary Institute of Medical Engineering of Fuzhou University, Fuzhou, Fujian, China; 5 Department of Radiation Oncology, The Second Affiliated Hospital of Fujian Medical University, Quanzhou, Fujian, China; 6 Department of Pathology, Clinical Oncology School of Fujian Medical University, Fujian Cancer Hospital, National Health Commission (NHC) Key Laboratory of Cancer Metabolism, Fuzhou, China

**Keywords:** lymph node metastasis, neoadjuvant immunochemotherapy, prognosis, recurrence pattern, thoracic esophageal squamous cell carcinoma

## Abstract

**Objective:**

To compare the patterns of lymph node metastasis (LNM) in patients with thoracic esophageal squamous cell carcinoma (TESCC) treated with neoadjuvant immunochemotherapy (nICT) versus neoadjuvant chemotherapy (nCT) alone and its impact on prognosis and potential clinical implications.

**Methods:**

A single-center retrospective cohort study was conducted on 441 patients with locally advanced TESCC who underwent nCT (n=179) or nICT (n=262) followed by esophagectomy. LNM patterns were analyzed according to the Japanese Classification of Esophageal Cancer (12^th^ Edition), using metrics including lymph node ratio (LNR), lymph node metastasis rate and actual lymph node metastasis rate for specific stations. Postoperative recurrence patterns, overall survival (OS), and event-free survival (EFS) were also evaluated.

**Results:**

Compared with nCT, nICT achieved a significantly higher pathological complete response (pCR) rate (22.1% vs. 6.7%, *p* < 0.001) and a lower metastatic lymph node ratio (LNR) (3.5% vs 6.2%, *p* < 0.001). Although the overall LNM rate was similar between groups, nICT demonstrated reduced lymph node involvement in several key station lymph nodes, particularly level 7 (along the left gastric artery), with lower the lymph node metastasis rate (LNMR2) (7.6% vs. 14.5%, *p* = 0.020) and the actual metastasis rate (LNMR3) (8.4% vs. 16.8%, *p* = 0.012). The overall recurrence/metastasis rate was significantly lower in the nICT group (36.2% vs. 56.8%, *p* < 0.001), with a notable reduction of recurrence at the anastomotic site. In multivariable analyses, nICT independently predicted lower recurrence risk (adjusted OR = 0.55, *p* = 0.013) and improved EFS (HR = 0.65, *p* = 0.001) while OS was not statistically different between groups.

**Conclusion:**

In comparison to nCT alone, nICT was significantly associated with deeper pathological response, lower LNM burden, and reduced postoperative recurrence in TESCC.

## 1 Introduction

Esophageal cancer (EC) remains one of the top 10 malignancies in China and worldwide (1), demonstrating a major health threat and social burden globally (2–4). Of note, the 5-year survival rate of EC in China is only about 27.9% (5). Quite different from the predominance of adenocarcinoma (EAC) (6) histology in Western countries, the majority of esophageal cancer cases in China are squamous cell carcinoma (ESCC), and this distinction dictates unique treatment strategies and prognostic characteristics (7–10).

Recently, treatment options for ESCC have diversified although surgery remains the primary curative modality. However, many patients were often present with regional lymph node metastasis (LNM) at the time of diagnosis, and were usually classified as locally advanced. LNM serves as a crucial prognostic indicator, and a higher ratio of metastatic lymph nodes often predicts poor prognosis (11–17). Currently, neoadjuvant chemotherapy (nCT) or neoadjuvant concurrent chemoradiotherapy (nCRT) plus surgery remains the standard-of-care for locally advanced ECs worldwide (18–21). However, with the development and prevalence of use of immune checkpoint inhibitors (e.g. PD-1/PD-L1 inhibitors), there has been ongoing debate regarding whether neoadjuvant immunochemotherapy (nICT) could be comparable with nCRT or nCT in yielding pathological complete response (pCR) rates and demonstrate non-inferior efficacy (22–24).

Although nICT significantly improves pCR rates, it remains unclear whether this systematic treatment alters the inherent patterns of LNM in ESCC. A majority of existing studies focused on overall nodal involvement or survival outcomes, while the specific impacts of neoadjuvant therapy on lymph node level-based metastasis patterns had been poorly defined. So, understanding these patterns is critically pivotal, as lymphatic drainage anatomy dictates both the extent of lymphadenectomy and postoperative surveillance strategies (9, 25, 26). Similarly, the impact of LNM on postoperative recurrence pattern remains unclear, either. Clarifying these patterns is crucial in guiding the precise extent of intraoperative lymph node dissection, identifying high-risk recurrence areas, and formulating individualized postoperative adjuvant treatment and follow-up strategies. Based on the aforementioned facts, we hypothesized that nICT followed by surgery would probably provide better outcomes compared with those receiving nCT plus surgery.

Our current study retrospectively analyzed these two patient cohorts and systematically compared the differences in LNM and postoperative recurrence patterns between groups. LNM patterns, including metastatic lymph node ratio and station-specific metastasis rates were compared. Postoperative recurrence patterns, overall survival (OS) and event-free survival (EFS) were also evaluated. Through this comprehensive analytical approach, our aim was to more precisely reveal the impact of neoadjuvant immunotherapy on the LNM burden and recurrence hallmarks of TESCC patients, and to provide evidence-based support for achieving more precise individualized comprehensive treatment.

## 2 Methods and materials

### 2.1 Study population and endpoints

**Figure 1:**
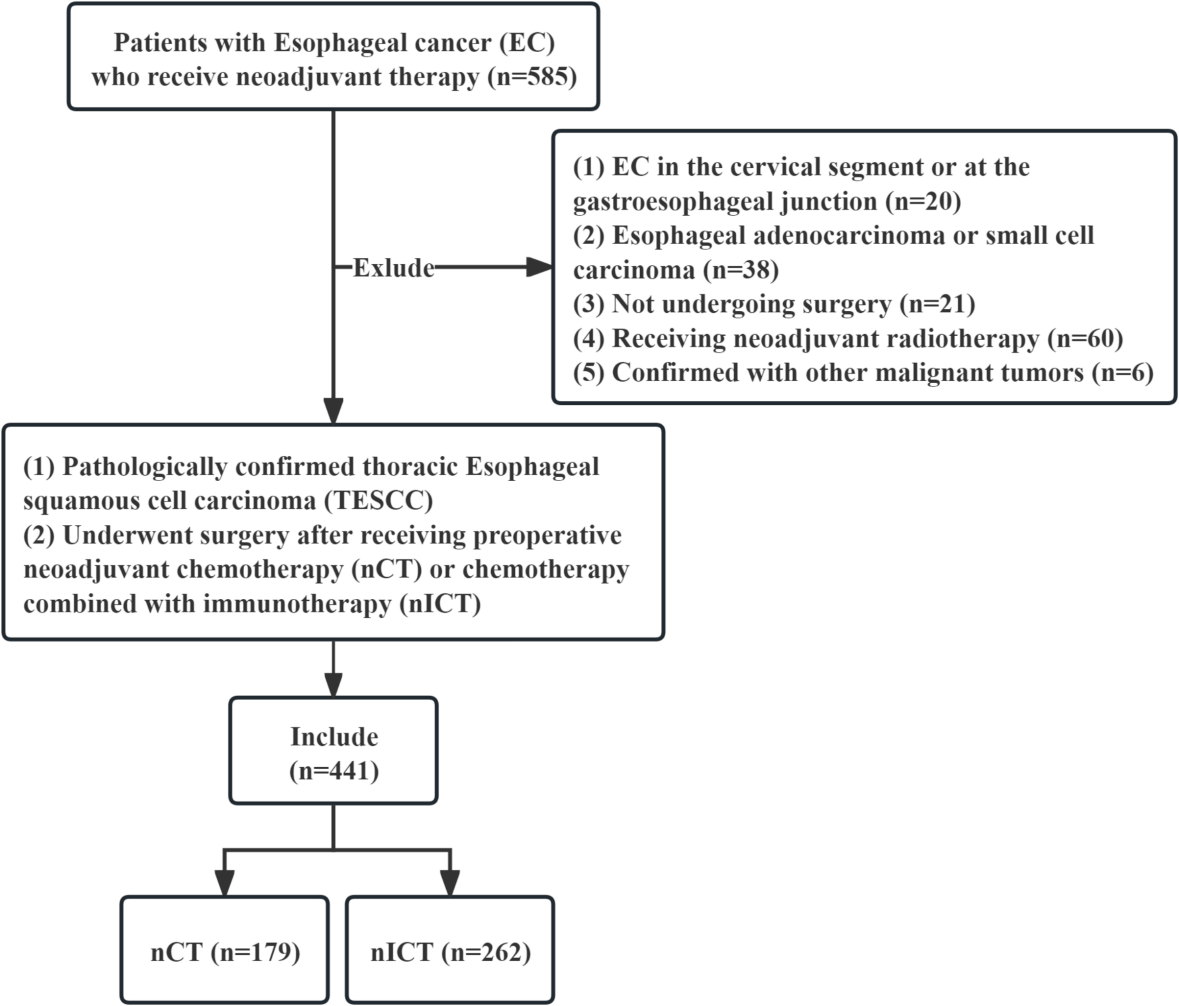
Research flowchart.

This is a single-center retrospective cohort study. Patients with TESCC who received nICT or nCT alone then followed by surgery in Fujian Medical University Cancer Hospital from January 2017 to December 2023 were screened and analyzed, as shown in the flowchart (**Figure 1**). The inclusion criteria were: (1) tumor locating in the thoracic esophagus; (2) histologically confirmed as ESCC; (3) underwent preoperative nICT or nCT; (4) underwent esophagectomy and lymph node dissection; and (5) absence of non-regional lymph node metastases and/or distant organ metastases, except for supraclavicular lymph node metastases. The exclusion criteria were: (1) cervical or esophagogastric junction ESCC; (2) esophageal adenocarcinoma, small cell carcinoma, or other histology; (3) received preoperative nCRT or other antitumor therapies; (4) simultaneously combined with other malignant tumors; and (5) with incomplete medical records. This study was approved by the Human Ethics Review Committee (Approval No.: K2025-340-01) of Fujian Medical University Cancer Hospital. The primary endpoint was LNM rate. Secondary endpoints included postoperative recurrence patterns, OS and EFS. OS was measured from the initiation of neoadjuvant therapy until death from any cause, whereas EFS was measured from the same start point to the first event among local recurrence, metastasis, or death from any cause.

### 2.2 Collection of clinicopathological data

Data were collected from the hospital’s medical record, including: gender, age, body mass index (BMI), personal medical history, esophageal tumor location, neoadjuvant treatment regimen, surgical data, pathological data, TNM stage, auxiliary examination data (complete blood count, biochemistry, endoscopy, CT, PET-CT, etc.), postoperative treatment, and follow-up data. Pretreatment clinical staging (cTNM) was determined using chest CT, PET-CT, and endoscopic ultrasound. TNM staging was based on the 8^th^ AJCC edition (27). Follow-up was conducted through outpatient reviews, inpatient records, and telephone inquiry.

### 2.3 Neoadjuvant regimens

All patients received at least one cycle of neoadjuvant therapy administered every 3 weeks (q3w) before surgery. The nCT consisted of taxane and platinum doublet agents. Taxanes included paclitaxel (150 mg/m² on day 1), nab-paclitaxel (260 mg/m² on day 1), or docetaxel (75 mg/m² on day 1). Platinum agents included cisplatin (75 mg/m² on day 1), carboplatin (AUC 5–6 on day 1), lobaplatin (30 mg/m² on day 1), or nedaplatin (80 mg/m² on day 1). For nICT, a PD-1 inhibitor was added to the taxane and platinum doublet agents. PD-1 inhibitors included pembrolizumab, camrelizumab, sintilimab and tislelizumab (each 200 mg intravenously on day 1, q3w), as well as toripalimab (240 mg intravenously on day 1, q3w) and serplulimab (300 mg intravenously on day 1, q3w). The specific agents were selected at the discretion of the treating physicians based on individual patient characteristics and clinical considerations.

### 2.4 Surgical approach and lymphadenectomy

**Figure 2:**
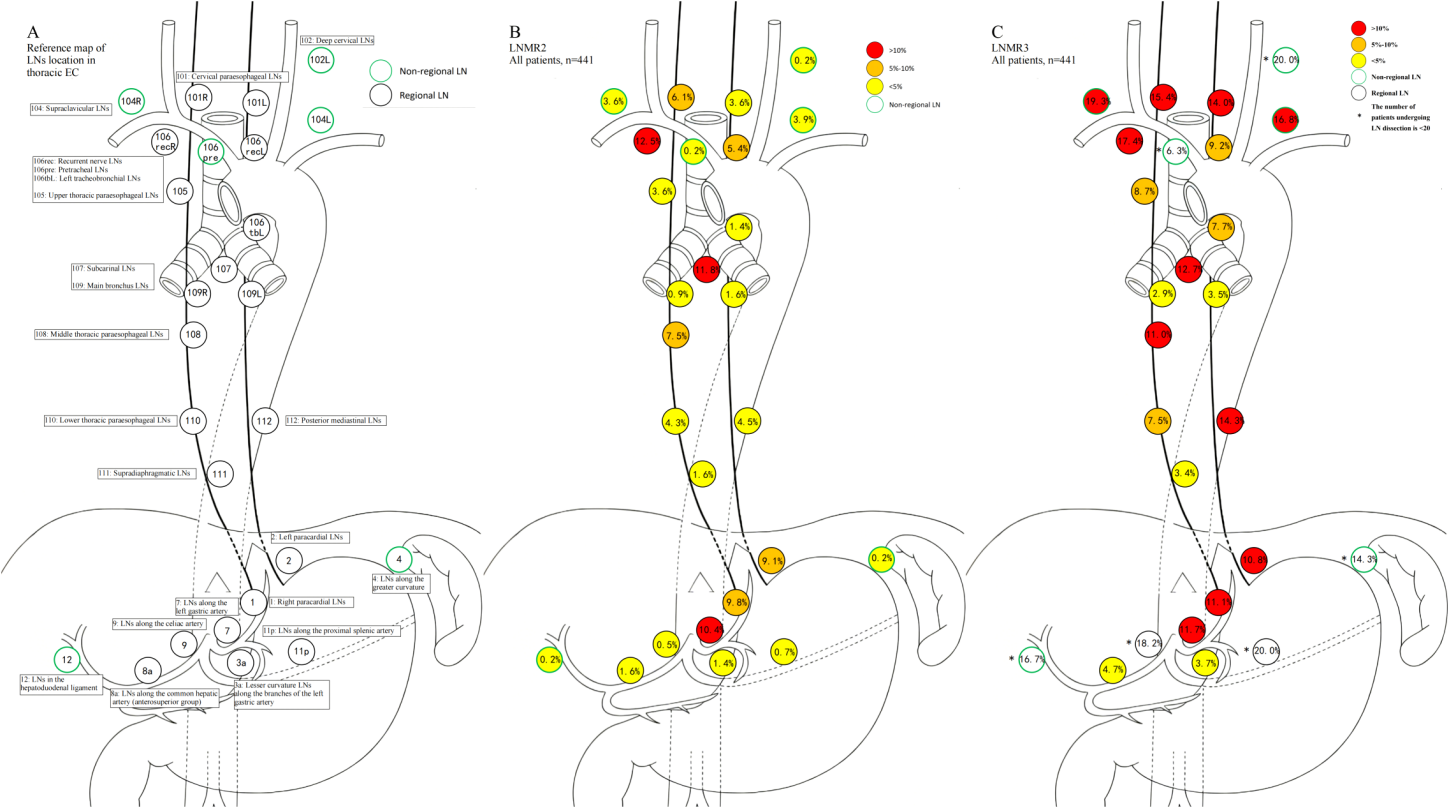
Schematic diagram of lymph node levels, LNMR2 and LNMR3 for All patients. **(A)** Schematic diagram of nodal levels in Japanese Classification of Esophageal Cancer (12^th^ Edition); **(B)** LNMR2 in all patients; **(C)** LNMR3 in all patients. The area within the black circle represents the lymph node levels of the thoracic esophageal squamous cell carcinoma, while the green circle represents the non-regional lymph node levels. Yellow, orange and red respectively represent the lymph node metastasis rates of < 5%, 5-10% and > 10%. In addition, “*” represents the number of people dissected at this lymph node level < 20.

All patients proceeded to esophagectomy with lymph node dissection 3–8 weeks after completion of the last neoadjuvant cycle. Surgical approach was categorized as open esophagectomy or minimally invasive esophagectomy (MIE). The operative procedure was further classified as McKeown (cervical anastomosis) or Ivor-Lewis (intrathoracic anastomosis) esophagectomy. The extent of lymphadenectomy was defined as two-field (thoracic and abdominal) or three-field (cervical, thoracic and abdominal) dissection. Cervical lymphadenectomy, when performed, included at minimum the cervical paraesophageal and supraclavicular nodes as defined by the Japan Esophageal Society (JES) nodal map (28). Lymph node stations were recorded according to the Japanese Classification of Esophageal Cancer (JES, 12^th^ edition) for cervical and thoracic stations (e.g., No.101-112), and abdominal nodal stations were documented following the Japanese gastric nodal station system adopted by the JES classification. The definitions of lymphadenectomy fields (two-field vs three-field) and the detailed lymph node station map are provided in **Figure 2A**, **Supplementary Table 1**. Resected tumor and lymph node specimens were independently evaluated by two pathologists (W. Xiao and Y. Shi).

### 2.5 Pathological assessment

Two senior pathologists evaluated the surgical specimens and issued the pathologic reports. Assessment included ypTNM stage, histological type, differentiation, circumferential resection margin (CRM), tumor regression, pathological response, immunohistochemistry and number of lymph nodes. Based on tumor regression, pathological complete response (pCR) was defined as the absence of viable tumor cells in both the primary site and resected lymph nodes (including carcinoma *in situ* at the primary site), and major pathological response (MPR) was defined as < 10% viable tumor cells in the primary tumor bed (29). R0 was defined as negative longitudinal resection margin and CRM, and R0 (close margin) was defined as negative longitudinal resection margin and tumor 0-1mm from CRM (30).

### 2.6 Lymph node metastasis patterns

Lymph node analysis and pattern summarization were conducted using the JES (28). To more comprehensively assess the true degree of lymph node involvement, we used the following metrics: ➊Lymph Node Ratio (LNR): Also known as the positive lymph node ratio, calculated as the total number of metastatic lymph nodes divided by the total number of resected lymph nodes (31); ➋Lymph Node Metastasis Rate (LNM_Rate): The proportion of patients with lymph node metastasis among the total patient population; ➌Actual Lymph Node Metastasis Rate (actual LNM_Rate): The proportion of patients with lymph node metastasis in a specific station among those who actually underwent dissection of that station; ➍For detailed analysis of specific lymph node stations, the following terms were defined: **I**. LNMR1_Level X = LNR for a specific level = Number of metastatic LNs in level X/Total number of LNs dissected from each patient; **II**. LNMR2_Level X = Lymph Node metastasis rate for level X = Number of patients with metastasis in level X/Total number of patients; **III**. LNMR3_Level X = Lymph Node actual metastasis rate for level X = Number of patients with metastasis in level X/Number of patients who actually had level X dissected. Based on previous studies (32), LNM rates were categorized into three levels: < 5%, 5-10%, and > 10%. Maps of metastasis rates for different stations were generated using yellow, orange, and red to represent these levels, respectively.

### 2.7 Tumor recurrence

Postoperative tumor recurrence was categorized as locoregional recurrence (LR) or distant recurrence (DR). LR was defined as recurrence at the esophagogastric anastomotic site or in regional lymph nodes (according to JES stations). DR was defined as tumor occurrence in other organs or non-regional lymph nodes. Recurrence or metastasis was determined based on pathological reports or comprehensive review of imaging findings. Specific positive imaging criteria included (1): higher avidity/uptake (SUVmax > 3.0) in PET-CT (2); irregular nodularity or irregular thickening > 5 mm with abnormal enhancement at the site of anastomosis in CT scanning (3); bulky, clustered or fused lymph nodes, or a short-axis diameter greater than > 10 mm in contrasted CT scanning (4); brain, bone, liver or pelvic metastases verified in combination with magnetic resonance imaging (MRI) (33, 34). To quantify the recurrence/metastasis burden, we established a scoring system: 1 point for a single LR site; 2 points for a single DR site; a total score of 8 points if the number of recurrence/metastasis sites was ≥ 4.

### 2.8 Statistical analyses

To reduce the influence of potential confounders in the two groups, we used propensity-score matching (PSM). We matched the two groups according to the 1:1 optimal matching principle with the use of the MatchIt package of R software. The characteristics of the two groups of patients were included for propensity score matching method, including age, gender, smoking, drinking, tumor location, clinical TNM stage, number of neoadjuvant therapy cycles, surgical approach, type of surgery, extent of resection, number of lymph node dissection and postoperative adjuvant therapy. Multiple statistical measures were used, with median (interquartile range) for continuous variables and frequency (percentage) for categorical variables. Categorical variables were analyzed with the use of the chi-square test or Fisher’s exact test, and continuous variables were compared with the use of the Wilcoxon rank-sum test or the Kruskal-Wallis test. Subgroup analyses were performed to assess the association between treatment group and pCR across different strata. Prespecified subgroup analyses were performed by number of neoadjuvant cycles, taxane and platinum doublet agent. Within each subgroup, multivariable logistic regression estimated adjusted ORs (95% CIs) for pCR comparing nICT with nCT, adjusting for sex, age, BMI, tumor location, cT, and cN. Treatment-by-subgroup interactions were tested and summarized in a forest plot. A receiver operating characteristic (ROC) curve was used to determine the optimal LNR cut-off using the Youden Index. Univariate logistic or Cox Regression analyses were first performed, followed by multivariate models including variables with *p* < 0.1. Kaplan-Meier curves were generated, and survival difference analysis was assessed using the Log-rank test. Statistical significance was defined as *p* < 0.05 (two-sided). Analyses were conducted using R software (Version 4.2.2).

## 3 Results

### 3.1 Patient baseline characteristics and subgroup analyses

As shown in **Table 1**, a total of 441 eligible TESCC patients were included in our study. The overall male-to-female ratio was 3.5:1, with a median age of 61 years old (range: 44–78 y.o.). Amongst them, 179 (40.6%) received nCT, and 262 (59.4%) received nICT. In comparison to nCT, nICT was associated with higher odds of pCR (12/179 [6.7%] vs 58/262 [22.1%], with an adjusted OR of 4.08, 95% CI 2.09-7.97; *p* < 0.001). The association was most predominant in patients receiving two cycles of nCT (adjusted OR 4.61, 95% CI 2.17-9.77; *p* < 0.001), with no significant interaction with number of cycles, taxane or platinum agent (*p* for interaction = 0.171, 0.108, and 0.093, respectively) (**Supplementary Figure 1**). In addition, **Table 1** still provided a comprehensive comparison of baseline characteristics between nCT group and nICT group before and after PSM. Baseline characteristics were well-balanced within groups (*p* > 0.05). Detailed distributions of chemotherapy agents and PD-1 inhibitors are provided in **Supplementary Table 2**.

**Table 1:** Patient baseline characteristics before and after propensity score matching (PSM).

Characteristics	Unmatched			Matched		
	nICT^1^ N = 262	nCT^2^ N = 179	*P*-value^3^	nICT N = 179	nCT N = 179	*P*-value^3^
**Age**, Median (Q1, Q3)	61 (56, 67)	60 (55, 65)	0.130	60 (55, 66)	60 (55, 65)	0.997
**Male**, n (%)	210 (80.2%)	132 (73.7%)	0.113	142 (79.3%)	132 (73.7%)	0.212
**BMI**, Median (Q1, Q3)	21.66 (19.65, 23.74)	21.60 (19.95, 23.72)	0.987	21.67 (19.49, 23.74)	21.60 (19.95, 23.72)	0.856
**Smoker**, n (%)	113 (43.1%)	71 (39.7%)	0.469	75 (41.9%)	71 (39.7%)	0.667
**Drinker**, n (%)	53 (20.2%)	35 (19.6%)	0.862	33 (18.4%)	35 (19.6%)	0.788
**Tumor Location**, n (%)			0.436			0.947
Upper	52 (19.8%)	28 (15.6%)		29 (16.2%)	28 (15.6%)	
Middle	121 (46.2%)	82 (45.8%)		84 (46.9%)	82 (45.8%)	
Lower	89 (34.0%)	69 (38.5%)		66 (36.9%)	69 (38.5%)	
**cT**^4^, n (%)			0.181			0.298
T1-2	46 (17.6%)	23 (12.8%)		30 (16.8%)	23 (12.8%)	
T3-4	216 (82.4%)	156 (87.2%)		149 (83.2%)	156 (87.2%)	
**cN**, n (%)			0.691			0.972
N0	38 (14.5%)	29 (16.2%)		30 (16.8%)	29 (16.2%)	
N1-2	209 (79.8%)	137 (76.5%)		137 (76.5%)	137 (76.5%)	
N3	15 (5.7%)	13 (7.3%)		12 (6.7%)	13 (7.3%)	
**cM**, n (%)			0.090			>0.999
M0	254 (96.9%)	178 (99.4%)		179 (100%)	178 (99.4%)	
M1 (Lymph node)	8 (3.1%)	1 (0.6%)		0 (0%)	1 (0.6%)	
**Neoadjuvant treatment cycles**, Median (Q1, Q3)	2.00 (2.00, 2.00)	2.00 (2.00, 2.00)	0.002	2.00 (2.00, 2.00)	2.00 (2.00, 2.00)	0.559
**Surgical approach**, n (%)			0.369			0.706
Ivor-Lewis	7 (2.7%)	9 (5.0%)		5 (2.8%)	9 (5.0%)	
McKeown	254 (96.9%)	169 (94.4%)		173 (96.6%)	169 (94.4%)	
Sweet	1 (0.4%)	1 (0.6%)		1 (0.6%)	1 (0.6%)	
**Surgical type**, n (%)			<0.001			0.090
Minimally Invasive Esophagectomy (MIE)	220 (84.0%)	126 (70.4%)		140 (78.2%)	126 (70.4%)	
Open or Conversion to Open	42 (16.0%)	53 (29.6%)		39 (21.8%)	53 (29.6%)	
**Resection degree**, n (%)			0.653			0.701
R0	221 (84.4%)	157 (87.7%)		152 (84.9%)	157 (87.7%)	
R0(close margin)	33 (12.6%)	18 (10.1%)		21 (11.7%)	18 (10.1%)	
R1	8 (3.0%)	4 (2.2%)		6 (3.4%)	4 (2.2%)	
**Number of lymph nodes dissected,** Median (Q1, Q3)	36 (26, 47)	26 (17, 35)	<0.001	32 (23, 39)	26 (17, 35)	<0.001
**Number of metastatic lymph node,** Median (Q1, Q3)	0.00 (0.00, 2.00)	0.00 (0.00, 1.00)	0.083	0.00 (0.00, 2.00)	0.00 (0.00, 1.00)	0.071
**Post_treatment**, n (%)	169 (64.5%)	125 (69.8%)	0.244	119 (66.5%)	125 (69.8%)	0.496
**ypT**^5^, n (%)			-			-
T0	47 (17.9%)	10 (5.6%)		29 (16.2%)	10 (5.6%)	
Tis	10 (3.8%)	2 (1.1%)		17 (9.5%)	12 (6.7%)	
T1	28 (10.7%)	12 (6.7%)		40 (22.3%)	24 (13.4%)	
T2	52 (19.8%)	24 (13.4%)		84 (46.9%)	95 (53.1%)	
T3	117 (44.7%)	95 (53.1%)		4 (2.2%)	30 (16.8%)	
T4a	8 (3.1%)	30 (16.8%)		0 (0%)	6 (3.4%)	
T4b	0 (0%)	6 (3.4%)		5 (2.8%)	2 (1.1%)	
**ypN**, n (%)			0.061			0.045
N0	150 (57.3%)	89 (49.7%)		101 (56.4%)	89 (49.7%)	
N1	72 (27.5%)	44 (24.6%)		53 (29.6%)	44 (24.6%)	
N2	34 (13.0%)	39 (21.8%)		20 (11.2%)	39 (21.8%)	
N3	6 (2.3%)	7 (3.9%)		5 (2.8%)	7 (3.9%)	
**ypM**, n (%)			0.823			0.534
M0	243 (92.7%)	165 (92.2%)		168 (93.9%)	165 (92.2%)	
M1 (Lymph node)	19 (7.3%)	14 (7.8%)		11 (6.1%)	14 (7.8%)	
**Tumor response**, n (%)			<0.001			<0.001
pCR	58 (22.1%)	12 (6.7%)		36 (20.1%)	12 (6.7%)	
MPR	41 (15.6%)	10 (5.6%)		26 (14.5%)	10 (5.6%)	
**Lymph node metastasis (LNM)**, n (%)			0.228			0.204
No	147 (56.1%)	90 (50.3%)		78 (43.6%)	90 (50.3%)	
Yes	115 (43.9%)	89 (49.7%)		101 (56.4%)	89 (49.7%)	
**Lymph node ratio (LNR)**, number (%)			<0.001			<0.001
Number of metastatic lymph node	354 (3.5%)	311 (6.2%)		237 (4.2%)	311 (6.2%)	
Number of lymph nodes without metastasis	9663 (96.5%)	4711 (93.8%)		5462 (95.8%)	4711 (93.8%)	
**LNR**, n (%)			<0.001			0.004
High	65 (24.8%)	75 (41.9%)		49 (27.4%)	75 (41.9%)	
Low	197 (75.2%)	104 (58.1%)		130 (72.6%)	104 (58.1%)	

^1^nICT, neoadjuvant immunochemotherapy; ^2^nCT, neoadjuvant chemotherapy; ^3^Wilcoxon rank sum test, Pearson’s Chi-squared test; Fisher’s exact test; ^4^cT, cN and cM refer to the clinical TNM stage before neoadjuvant therapy. ^5^yPT, ypN and ypM refer to the postoperative TNM stage after neoadjuvant therapy.

Bold text indicates variable category subheadings used for table structure only; it does not denote statistical significance or any special annotation.

### 3.2 Overall lymph node metastasis status

Postoperative pathologic evaluation confirmed a LNM rate of 46.3% (204/441) for the entire cohort, with no statistical difference between nCT and nICT groups (49.7% vs. 43.9%, *p* = 0.228). However, regarding LNR, the nICT group showed a significant advantage, with a significantly lower LNR than that of the nCT group before (3.5% vs. 6.2%, *p* < 0.001) and after (4.2% vs. 6.2%, *p* < 0.001) matching. ROC curve analysis indicated that LNR had good predictive value for OS, with an AUC of 0.675 (95% CI: 0.629-0.720). The optimal LNR cut-off value was 0.041, as was determined by the maximum Youden index. Based on this cut-off value, LNR was dichotomized into High (≥ 0.041) and Low (< 0.041) subgroups. In the nICT group, the proportion of patients with High LNR was significantly lower than in the nCT group (24.8% vs. 41.9%, *p* < 0.001). Detailed lymph node dissections and metastasis data are provided in **Supplementary Table 3**.

### 3.3 Lymph node metastasis patterns

**Figure 3:**
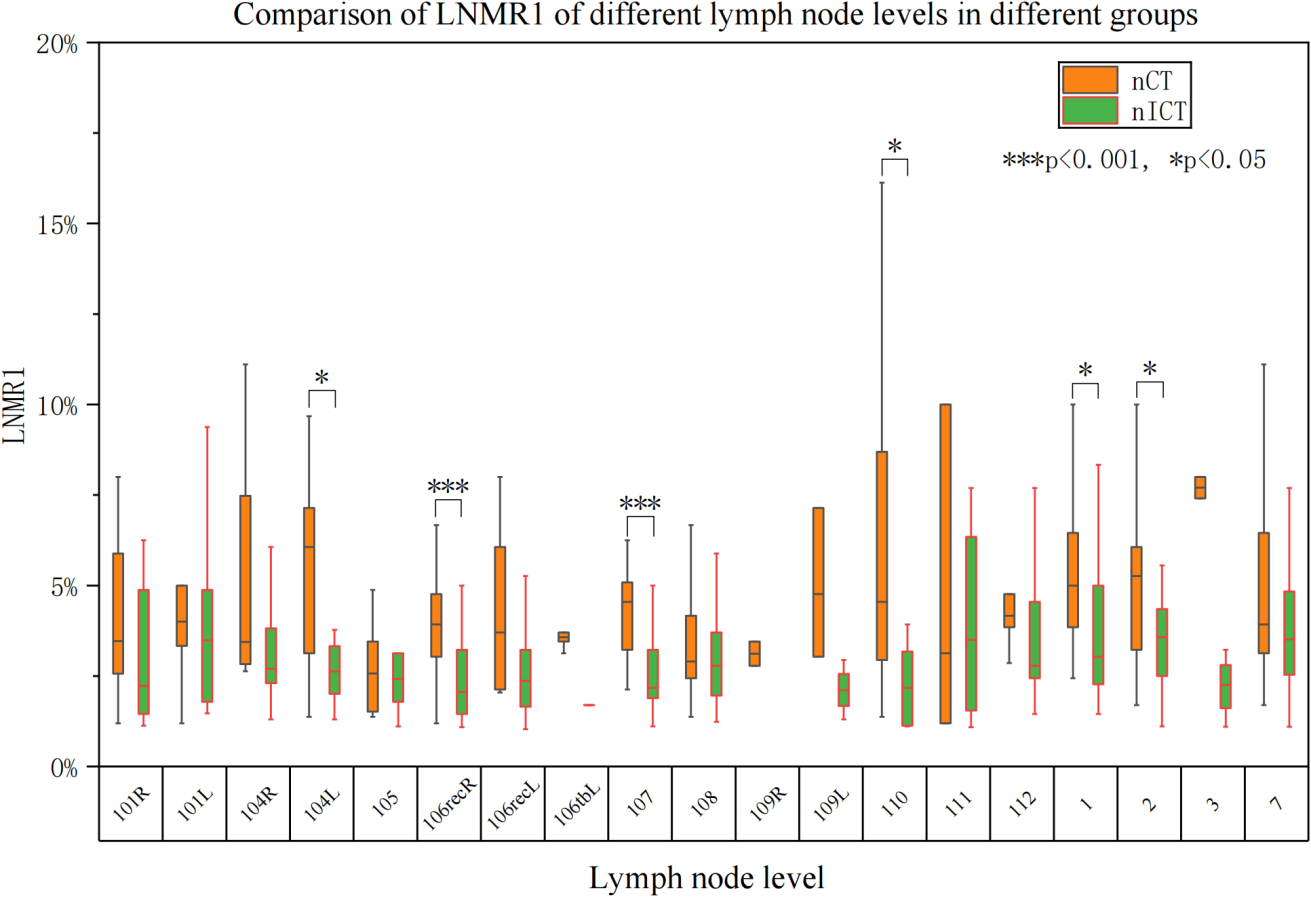
Comparison of lymph node ratio (LNR/LNMR1). This figure compares the median LNMR1 between neoadjuvant chemotherapy (nCT) and neoadjuvant immunochemotherapy (nICT). Orange represents nCT group and green represents nICT group. The horizontal axis represents the metastatic lymph node ratio and the vertical axis represents different lymph node levels. “*” indicates significant differences between the two groups. *P < 0.05, **P < 0.01, ***P < 0.001.

**Figure 4:**
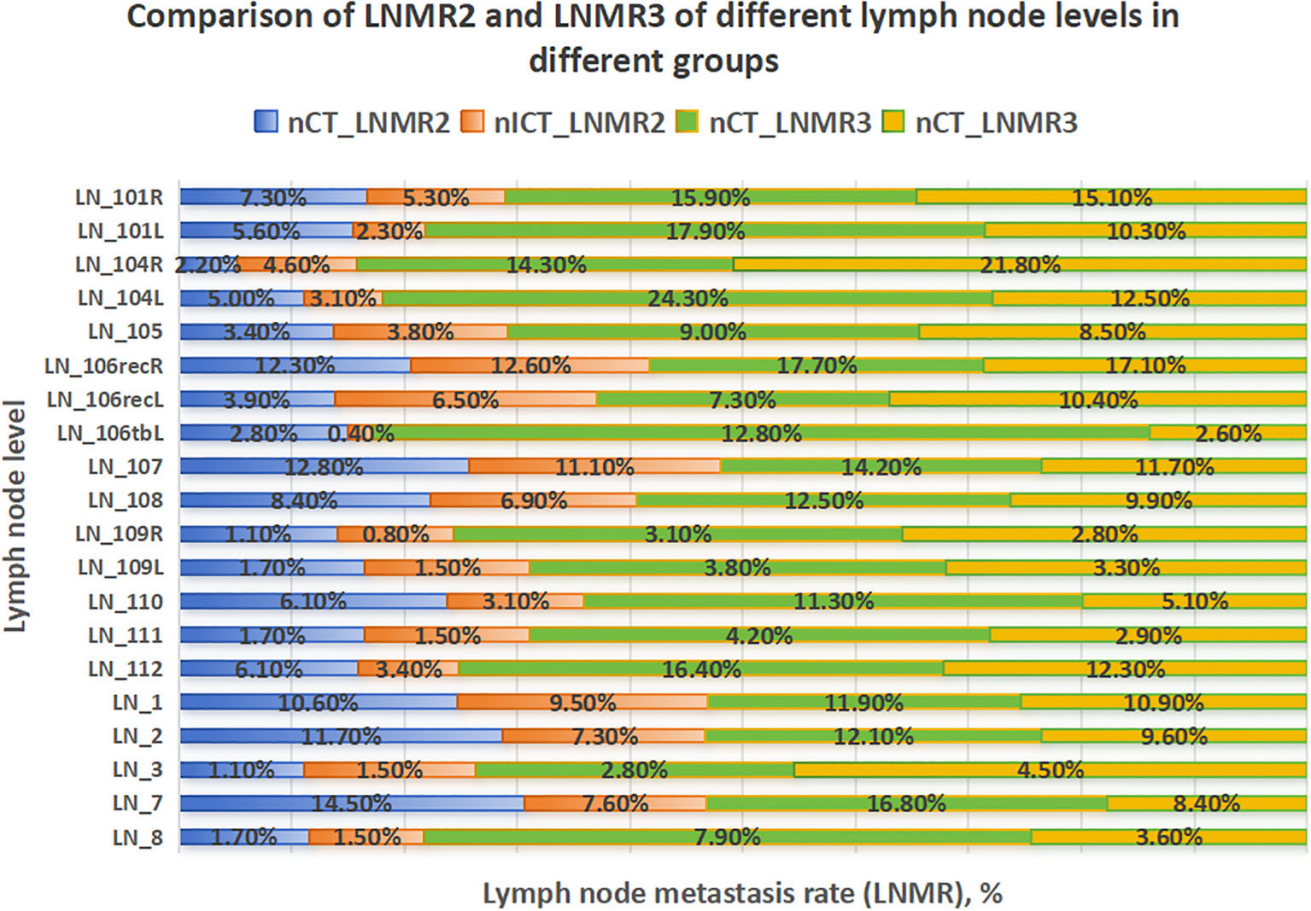
Comparison of LNMR2 and LNMR3. This figure compares the LNMR2 and LNMR3 between neoadjuvant chemotherapy (nCT) and neoadjuvant immunochemotherapy (nICT). The blue and red bar graphs on the left represent two groups of LNMR2 respectively. And the green and yellow bar graphs on the right represent the LNMR3 of the two groups. The vertical axis represents different lymph node levels.

**Figure 5:**
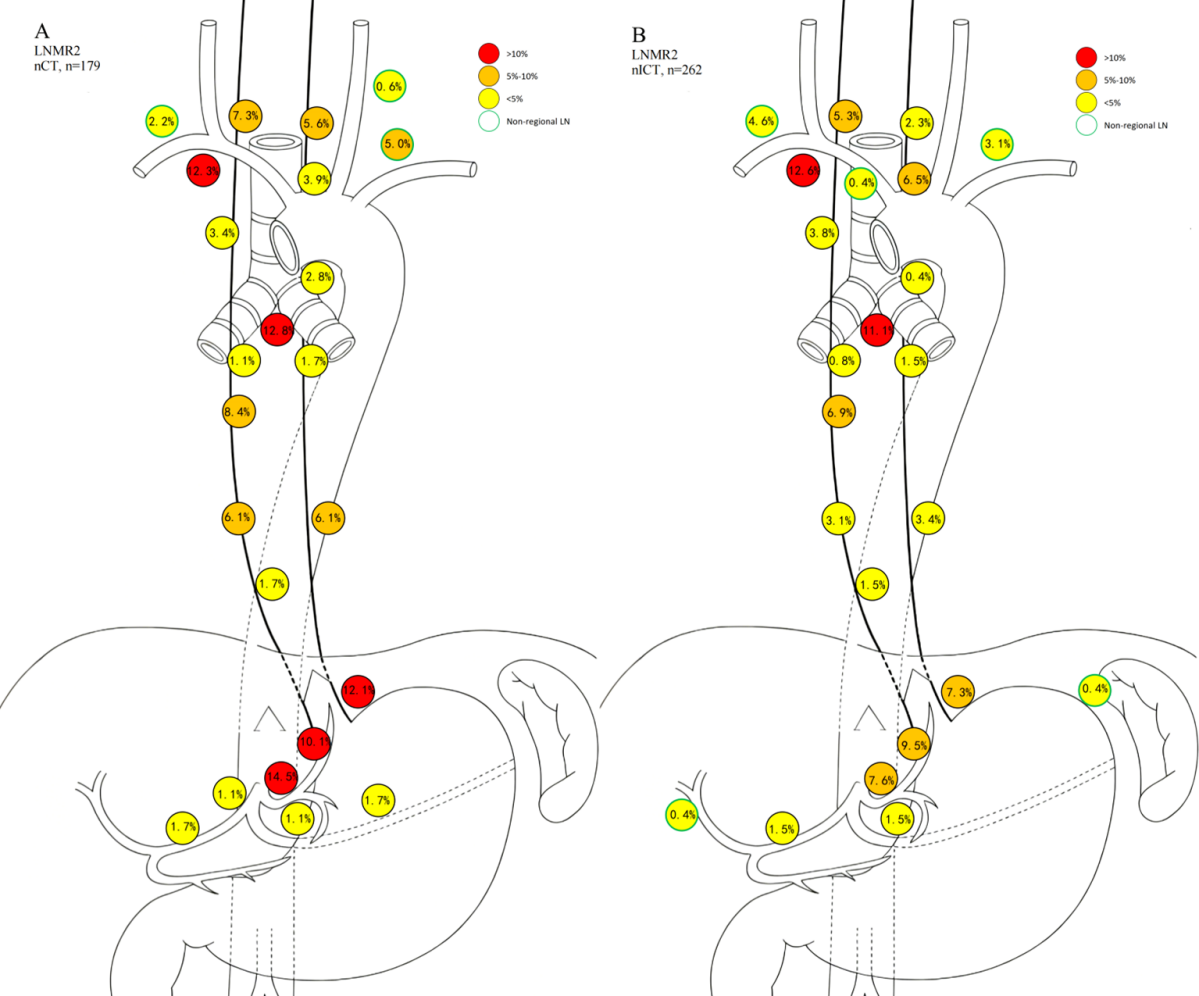
Schematic diagram of LNMR2 for nCT and nICT. **(A)** nCT (neoadjuvant chemotherapy); **(B)** neoadjuvant immunochemotherapy (nICT).

**Figure 6:**
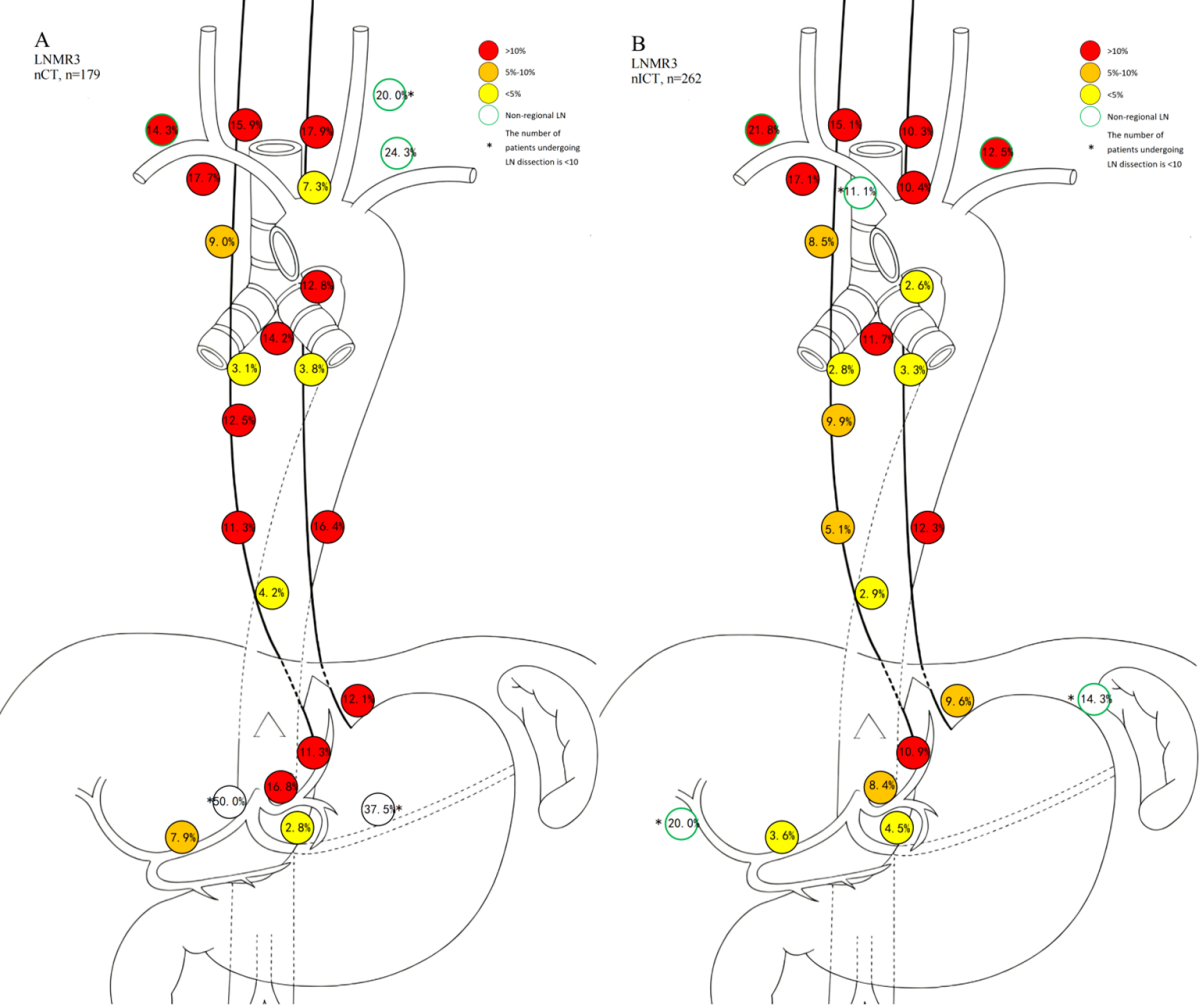
Schematic diagram of LNMR3 for nCT and nICT. **(A)** nCT (neoadjuvant chemotherapy); **(B)** neoadjuvant immunochemotherapy (nICT).

Heatmaps illustrating lymph node level-specific metastasis rates were shown in **Figures 2B, C**, **3**–**6**. There were clear station-specific differences in lymph node metastasis patterns between groups (**Figures 3**-**6**). Further analysis of lymph node metastasis patterns revealed that nICT group had significantly lower LNMR1 than that in nCT group regarding several key lymph node stations, including level 104L, 106recR, 107, 110, 1 and 2 (all *p* < 0.05), as illustrated in **Figure 3**. Importantly, level 7 (lymph nodes along the left gastric artery) was a common metastatic hotspot in the nCT group. Both the lymph node metastasis rate (LNMR2: 7.6% vs. 14.5%, *p* = 0.020) and the actual metastasis rate (LNMR3: 8.4% vs. 16.8%, *p* = 0.012) for level 7 were significantly lower in the nICT group, suggesting that nICT would specifically influence the lymphatic metastasis pathway of ESCC. Furthermore, the number of stations with LNMR2 and LNMR3 greater than 10% was higher in the nCT group than that in the nICT group. In nCT and nICT groups, the highest LNMR2 values were for level 107 (12.8%) and 106recR (12.6%), respectively, and the highest LNMR3 values were for level 104L (24.3%) and 104R (21.8%), respectively. Metastasis rates for different lymph node levels in each group were showed in **Figure 4**. After matching, nICT group had a lower median LNMR1 at level 2 (0.036 vs. 0.053, *p* = 0.027), and there was no significant difference in LNMR2 and LNMR3 between the two groups. Complete lymph node metastasis data were provided in **Supplementary Tables 4-6**.

### 3.4 Postoperative recurrence patterns

Until September 1, 2025, 7 patients (1.6%) were lost to follow-up (3 in nCT group, 4 in nICT group). The median follow-up time was 5.97 years in nCT group and 3.16 years in nICT group. Survival analyses indicated that nICT would significantly improve EFS (HR = 0.65, 95% CI: 0.50-0.84, *p* = 0.001). Although nICT demonstrated a trend towards better OS, the difference was not statistically significant (HR = 0.90, 95% CI: 0.69-1.17, *p* = 0.436). Regarding the recurrence patterns, the overall recurrence/metastasis rate was significantly lower in the nICT group than that in the nCT group (36.2% vs. 56.8%, *p* < 0.001), and the median recurrence/metastasis score was lower (*p* < 0.001) too. Mediastinal LNM and anastomotic site recurrence were the main recurrence patterns. nICT significantly reduced the risk of anastomotic site recurrence (9.6% vs. 18.2%, *p* = 0.009). Supraclavicular lymph nodes were the most common site of non-regional LNM, and lung, bone, and liver were the most common organs of distant metastases, as shown in **Table 2**.

**Table 2:** Recurrence patterns of TESCC patients treated with nCT or nICT.

Characteristic	Group		*P*-value
	nCT N = 179	nICT N = 262	
**Recurrence or/and metastasis, n (%)**	100 (56.8%)	94 (36.2%)	<0.001^2^
**Recurrent or/and metastatic status, n (%)**			<0.001^2^
No	76 (43.2%)	166 (63.8%)	
LR	39 (22.2%)	32 (12.3%)	
DR	26 (14.8%)	20 (7.7%)	
LR + DR	35 (19.9%)	42 (16.2%)	
**Recurrent or/and metastatic score, Median (Q1, Q3)**	1.00 (0.00, 3.00)	0.00 (0.00, 2.00)	<0.001^1^
**Location of LR**			
Anastomotic	32 (18.2%)	25 (9.6%)	0.009^2^
Mediastinal	48 (27.3%)	51 (19.6%)	0.061^2^
Cervical (LN_101)	2 (1.1%)	1 (0.4%)	-^3^
**Location of DR**			
Lung	15 (8.5%)	20 (7.7%)	-
Bone	12 (6.8%)	22 (8.5%)	-
Liver	10 (5.7%)	13 (5.0%)	-
Pleura	5 (2.8%)	6 (2.3%)	-
Brain	5 (2.8%)	5 (1.9%)	-
Peritoneum	7 (4.0%)	3 (1.2%)	-
Trachea	3 (1.7%)	4 (1.5%)	-
Others_1^4^	6 (3.4%)	10 (3.8%)	
**Nonregional lymph nodes**			
Supraclavicular	21 (11.9%)	27 (10.4%)	-
Retroperitoneal	16 (9.0%)	13 (5.0%)	-
Others_2^5^	3 (1.7%)	5 (1.9%)	-

^1^Wilcoxon rank sum test; ^2^Pearson’s Chi-squared test; ^3^”-” indicates that the difference test was not performed or the number of cases was too small. ^4^Others_1 include thyroid gland, muscle, renal, adrenal gland and spleen; ^5^Others_2 include axillary, pelvic cavity and inguinal lymph nodes.

Bold text indicates variable category subheadings used for table structure only; it does not denote statistical significance or any special annotation.

**Figure 7:**
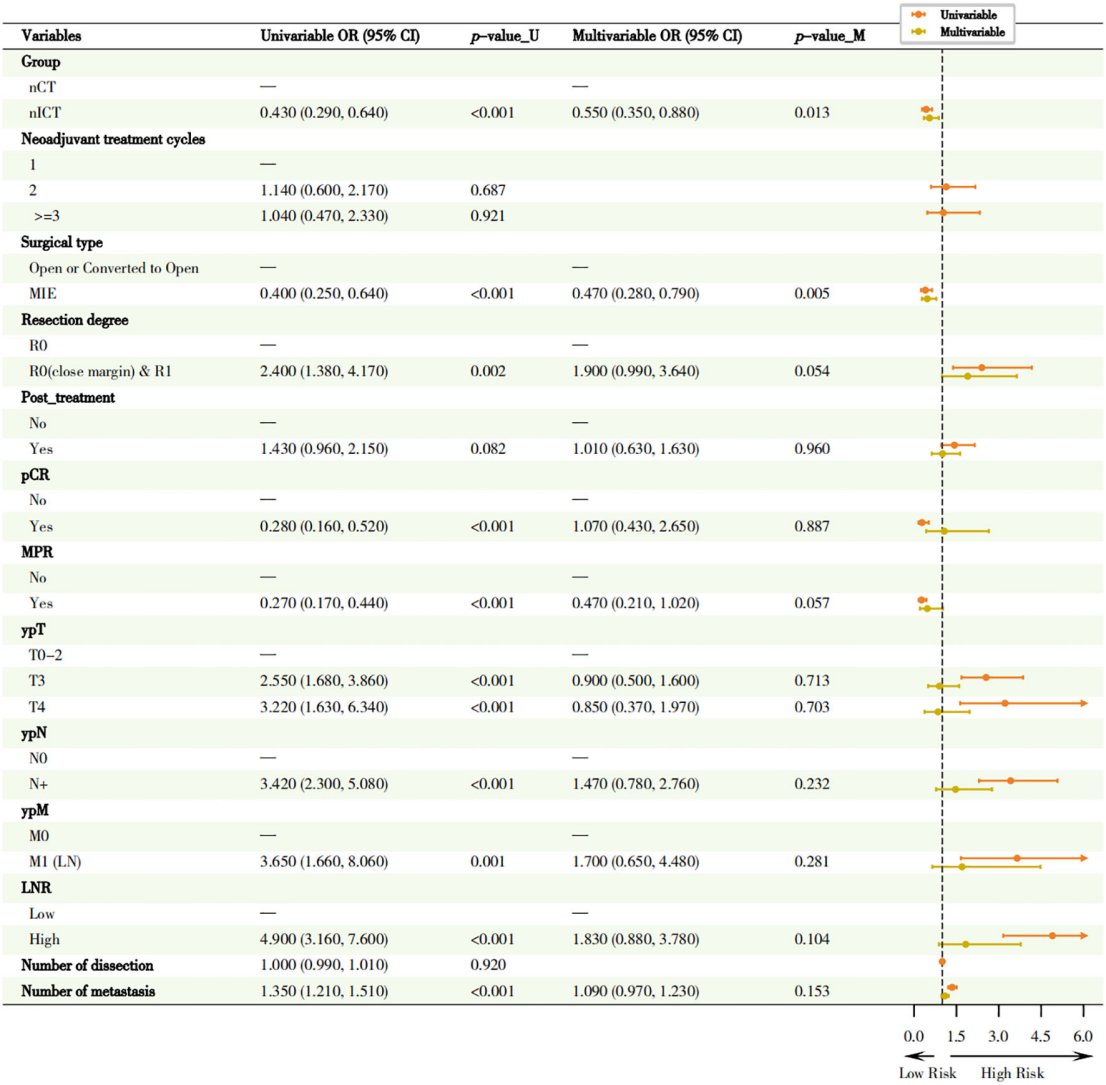
Forest plot of univariate and multivariate analyses of recurrence/metastasis (Logistic regression). This figure presents the clinical pathological factors identified through univariate and multivariate Logistic regression analysis, which are associated with postoperative recurrence/metastasis in patients. Circles located to the left of the dotted line indicate that the factor is a protective factor (OR < 1), while those located to the right indicate it is a risk factor (OR > 1).

As shown in **Figure 7**, multivariate logistic regression analysis showed that patients receiving nICT had a significantly lower risk of recurrence/metastasis compared to those receiving nCT alone (adjusted OR = 0.55, 95% CI: 0.35-0.88, *p* = 0.013). MIE significantly reduced the risk of recurrence or metastasis compared to open or converted open surgery (adjusted OR = 0.47, 95% CI: 0.28-0.79, *p* = 0.005). R0 (close margin) & R1 resection showed a trend toward higher risk of recurrence/metastasis (adjusted OR = 1.9, 95% CI: 0.99-3.64, *p* = 0.054), and achieving MPR showed a trend towards reduction of risk of recurrence/metastasis (adjusted OR = 0.47, 95% CI: 0.21-1.02, *p* = 0.057).

### 3.5 Survival analysis

**Figure 8:**
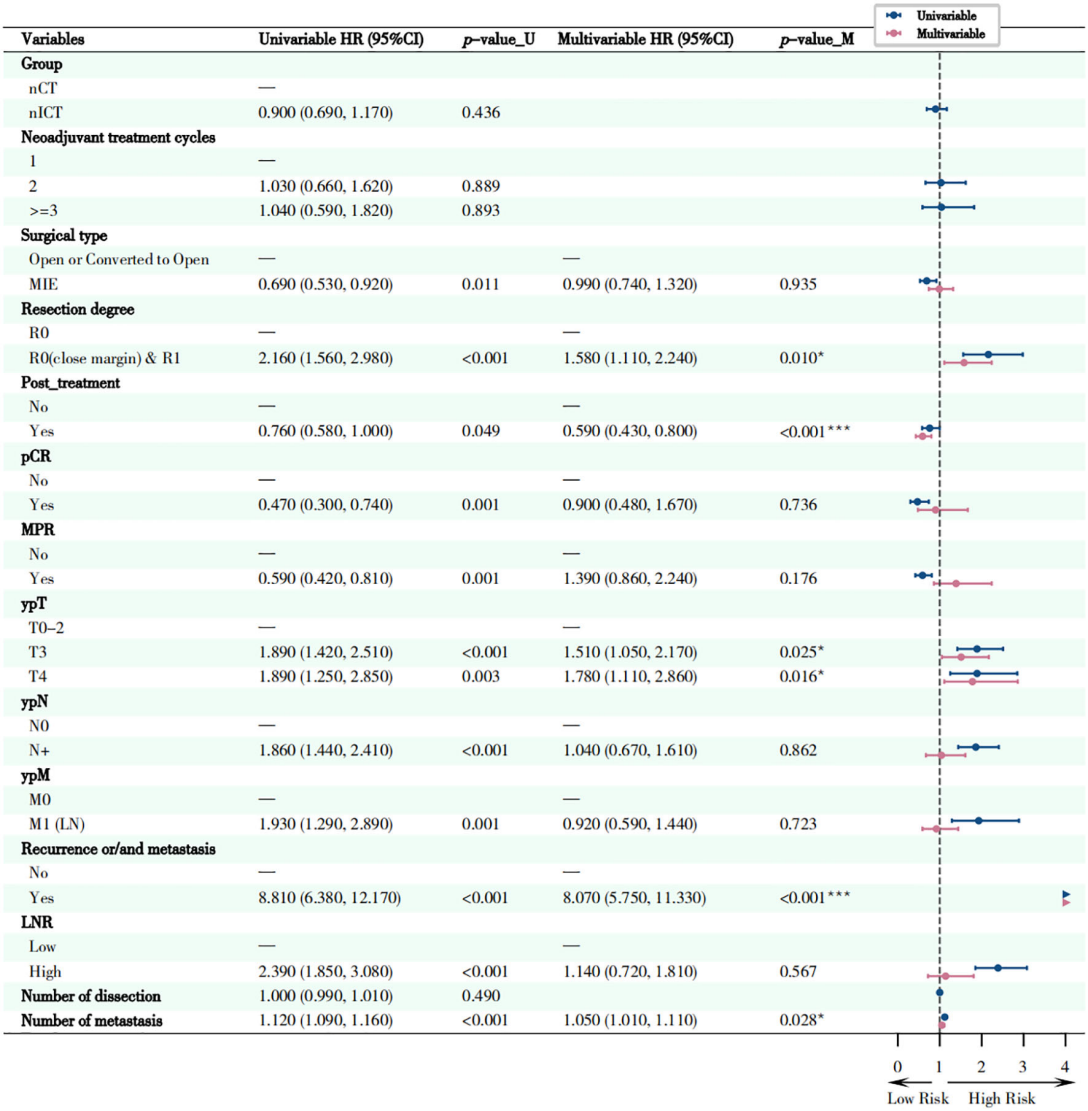
Forest plot of univariate and multivariate survival analysis of overall survival (OS). This figure presents the independent prognostic factors identified through univariate and multivariate Cox proportional hazards regression analysis, which are related to the overall survival (OS) of patients. Circles located to the left of the dotted line indicate that the factor is a protective factor (HR < 1), while those to the right indicate it is a risk factor (HR > 1). *P < 0.05, **P < 0.01, ***P < 0.001.

**Figure 9:**
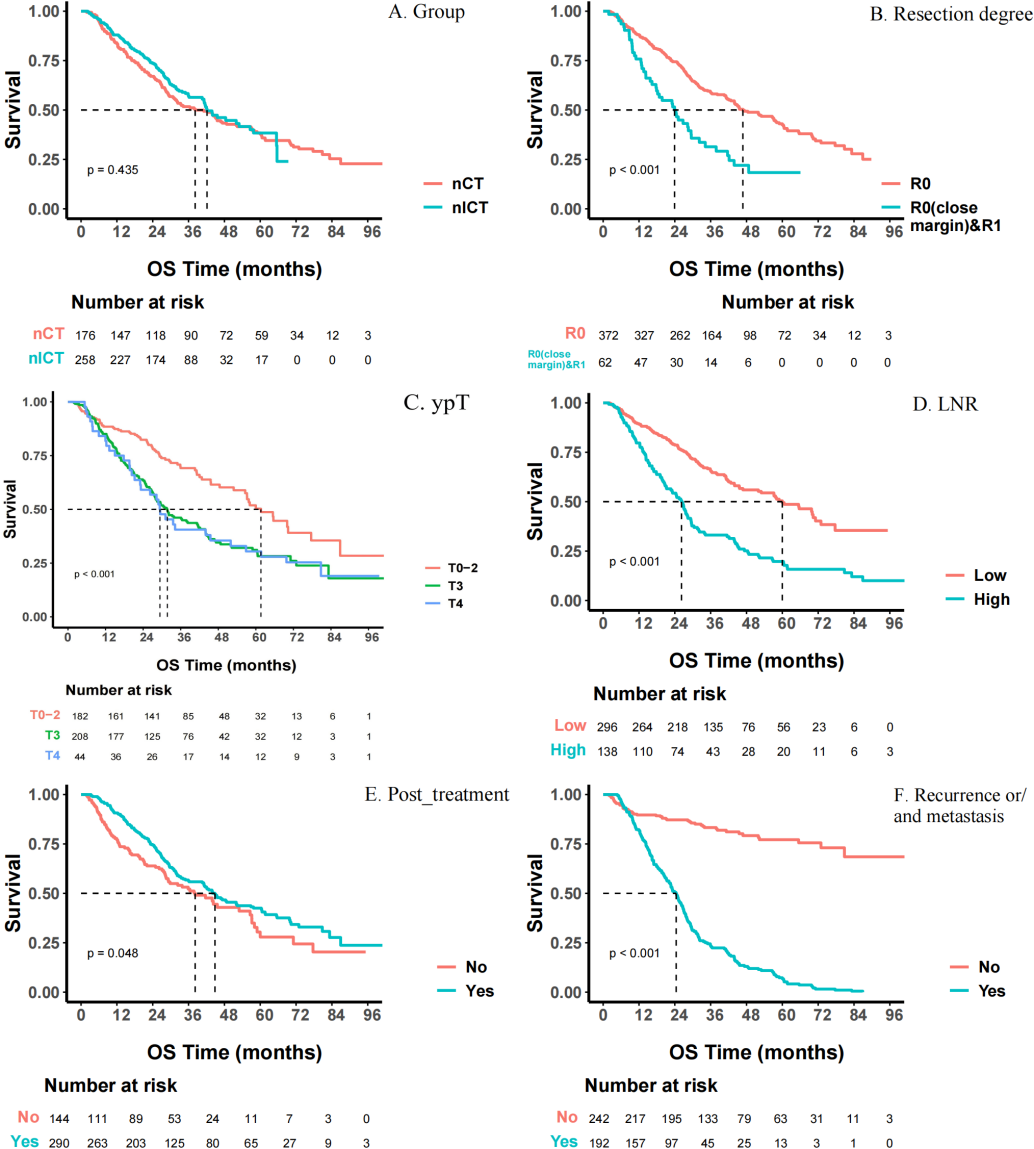
Kaplan-Meier curves for overall survival (OS) stratified by different factors. **(A)** Different methods of neoadjuvant treatments; **(B)** Resectibility; **(C)** Postoperative Pathological staging (ypT); **(D)** lymph node ratio (LNR); **(E)** Recurrence or/and metastasis; **(F)** Post_treatment. The survival differences among each group were evaluated using the Log-rank test.

As indicated in **Figure 8**, multivariate Cox regression analyses revealed several factors independently associated with OS. Firstly, non-R0 resection (including close margins and R1 resection), higher ypT stage (ypT3-4), and presence of postoperative recurrence/metastasis were factors significantly associated with higher risk of cancer-related death, especially postoperative recurrence/metastasis (HR = 8.07, 95%CI: 5.75-11.33, *p* < 0.001). However, receiving postoperative adjuvant therapy was an independent beneficiary factor (HR = 0.59, 95% CI: 0.43-0.80, *p* < 0.001) favoring longer survival. Kaplan-Meier survival curves for OS were shown in **Figure 9**.

**Figure 10:**
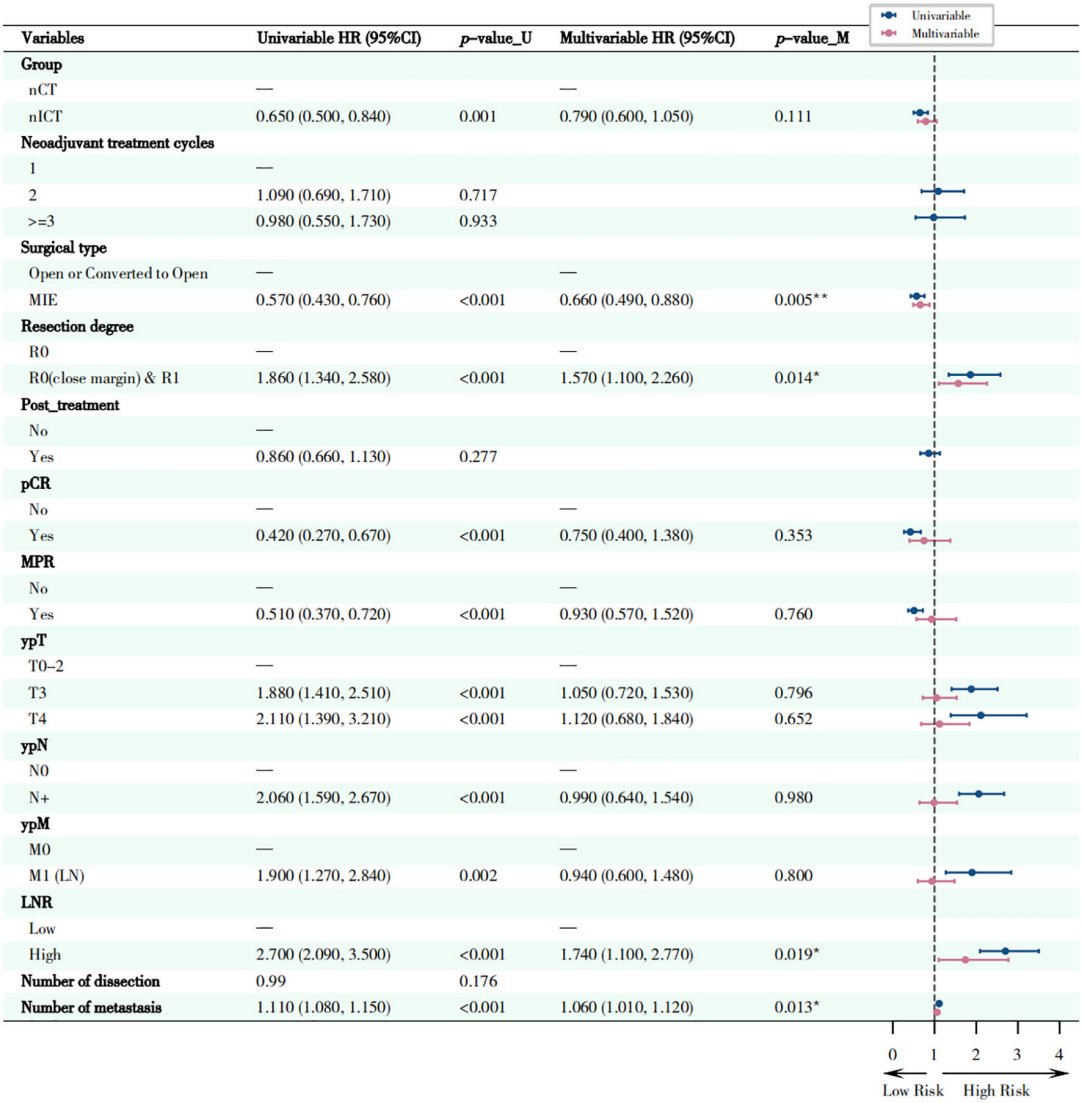
Forest plot of univariate and multivariate survival analysis of event-free survival (EFS). This figure presents the independent prognostic factors associated with event-free survival (EFS) of patients as determined by univariate and multivariate Cox proportional hazards regression analysis. Circles located to the left of the dotted line indicate that the factor is a protective factor (HR < 1), while those to the right indicate it is a risk factor (HR > 1). *P < 0.05, **P < 0.01, ***P < 0.001.

**Figure 11:**
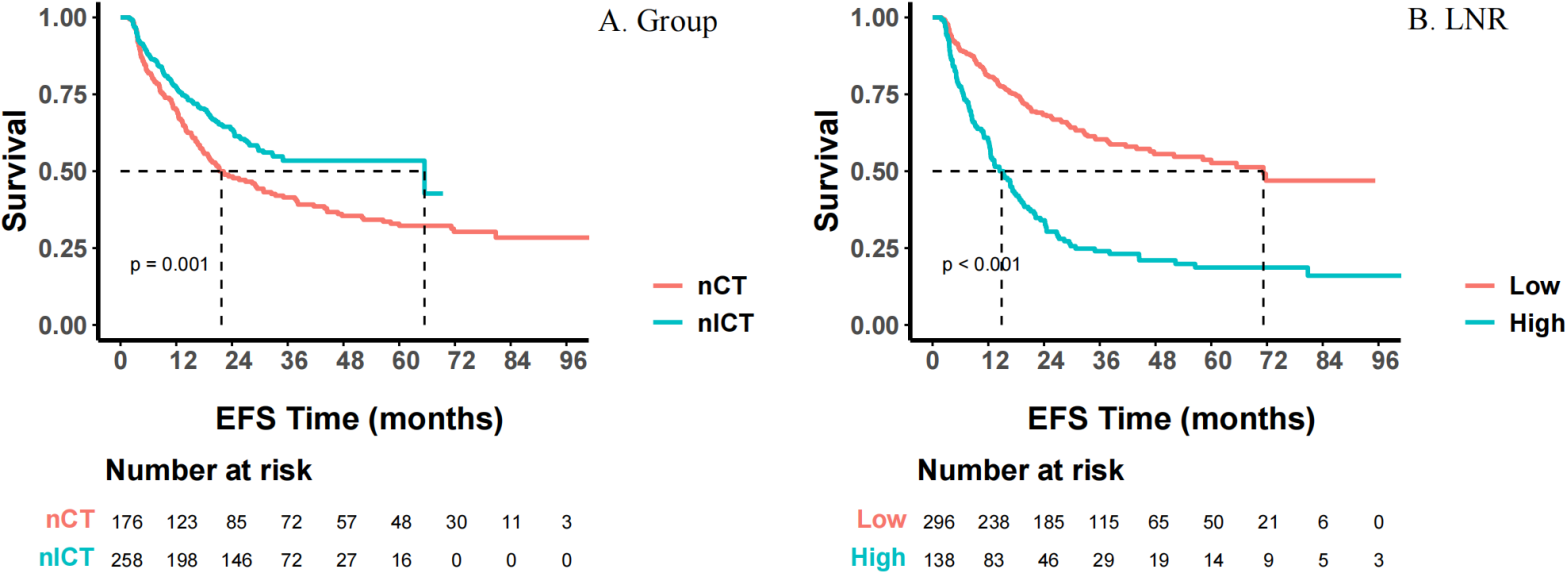
Event-free survival (EFS) curve. **(A)** Different groups based on different neoadjuvant modalities; **(B)** lymph node ratio (LNR). The survival differences among each group were evaluated using the Log-rank test.

As shown in **Figure 10**, multivariate Cox regression analysis showed that EFS was significantly associated with several factors. MIE significantly reduced the risk of events (adjusted HR = 0.66; 95% CI: 0.49-0.88; *p* = 0.005). R0 resection (close margin) & R1 resection (adjusted HR = 1.57; 95% CI: 1.10-2.26; *p* = 0.014) and high LNR (adjusted HR = 1.74; 95% CI: 1.10-2.77; *p* = 0.019) significantly increased the risk of events. For each scale increment in the number of metastatic LNs, the risk of events increased correspondingly (adjusted HR = 1.06; 95% CI: 1.01-1.12; *p* = 0.013). Corresponding Kaplan-Meier survival curves for EFS were shown in **Figure 11**.

## 4 Discussion

In this study, lymph node metastasis patterns and postoperative recurrence between patients with TESCC treated with nICT versus nCT alone were compared and analyzed. We found that, first, nICT was associated with significantly higher pathological response rate, as was consistent with the findings of the Randomized Controlled Trials (RCT) and real-world studies, demonstrating an enhancement on the anti-tumor effect with the presence of immune checkpoint inhibitors to the conventional chemotherapy regimen (35, 36). Second, nICT was associated with a markedly lower recurrence/metastatic rate and better EFS. Third, although the overall LNM rate did not significant difference between groups, the nICT group exhibited a lower LNR and reduced metastasis rates in select LN stations (station 7), suggesting a more effective systematic disease control.

While immune checkpoint inhibitors have been incorporated into Food and Drug Administration (FDA)-approved treatment paradigms for esophageal cancer in advanced or metastatic disease and in selected adjuvant settings (e.g., pembrolizumab for advanced disease; nivolumab as adjuvant therapy after resection), their use as neoadjuvant therapy for locally advanced ESCC remains under investigation (37, 38). Consequently, neoadjuvant concurrent chemoradiotherapy remains a widely accepted standard-of-care approach for appropriate patients. In this context, our institution recently compared neoadjuvant immunochemotherapy with chemoradiation, demonstrating a comparable pCR rate in both modalities (32.7% versus 34.6%) (24). In addition to this, recently, more attempts had been tried to compare the efficacy of nICT to that of nCRT or nICRT followed by surgery in the management of esophageal cancer (39–43). In consideration of the patient compliance and facilitation, researchers are always trying to demonstrate a non-inferior efficacy of nICT to other regimens including nCT and nCRT. However, in PALACE-2 study, the short-term efficacy of neoadjuvant pembrolizumab plus chemoratiotherapy was not superior to that of neoadjuvant chemoradiotherapy (42).

A major observation of this study was that nICT was correlated with lower LNR in several key lymph node stations (e.g. level 106recR, 107, 1 and 2) and lower lymph node metastasis rate (LNMR2) and actual lymph node metastasis rate (LNMR3) in station 7. Although these findings aligned with the hypothesis that immunotherapy might probably eradicate some suspicious micro-metastatic diseases, they should be interpreted cautiously (44). The nICT group underwent more extensive lymphadenectomy and a higher likelihood of MIE, both would influence LNMR values. Therefore, the observed difference would by some degree reflect surgical factors or selection patterns rather than intrinsic modification of lymphatic spread. To analyze the role of LNR, we conducted a further PSM analysis of the number of lymph node dissections in the two groups to obtain more reliable comparative results, finding that the number of dissected lymph nodes in the nICT group was still greater than that in the nCT group, which might lead to a larger denominator of LNR and data bias.

Consistent with improved pathological response and reduced lymph node metastasis rates, the overall incidence of recurrence/metastasis was significantly lower in the nICT group. Multivariate analysis further confirmed nICT as an independent protective factor. It is particularly noteworthy that nICT significantly reduced recurrence at anastomotic site, probably and potentially stemming from the higher R0 resection rate and more thorough local tumor clearance, which was ultimately converted into a significant improvement in EFS. It’s surprising to us all that no significant difference had ever been found in OS, as was probably owing to the relatively shorter median follow-up time in the nICT group. With extended follow-up, OS benefit would probably be expected, as had been precedented by other studies (44, 45).

A highlight of our study was the analysis of the actual metastasis rate (LNMR3) for each lymph node station, which excluded patients who did not undergo dissection of a particular station or had no lymph nodes found in that station upon pathological examination. This provided a more accurate delineation of station-specific nodal involvement and could serve as a reference standard for future research in lymph node involvement. The lymph node metastasis maps generated from actual clinical data provided valuable reference for studies on lymph node dissection. Additionally, we included patients with R0 (close margin) in the analyses. These patients as well as those with R1 resection experienced impacts on postoperative recurrence and survival, requiring closer surveillance and further analysis in subsequent studies. Our survival analyses re-emphasized the importance of R0 resection, postoperative adjuvant therapy, and effective control of recurrence and metastasis for prolonging OS (35). Furthermore, recurrence and survival were also related to individual patient differences, patient compliance, and other factors that need further consideration.

Although the AJCC/UICC staging system defines supraclavicular lymph node metastasis as M1, it is classified as M1a according to the Japanese Classification of Esophageal Cancer, 12^th^ Edition (applicable to thoracic esophageal and esophagogastric junction tumors), indicating that patients of this sort may still be eligible for radical surgery with lymphadenectomy (28). Classifying postoperative extra-regional lymph node metastasis (mainly supraclavicular) as M1 for relevant factor analysis might show significance in univariate analysis regarding postoperative recurrence/metastasis, OS, and EFS, even if not significant in multivariate analysis.

Biomarker stratification is clinically relevant when interpreting outcomes of immune checkpoint inhibition. In esophageal cancer, PD-L1 expression assessed by immunohistochemistry (commonly reported as CPS or TPS) and other emerging markers (e.g., MSI/MMR status and tumor mutational burden) have been explored for predicting response and guiding treatment selection (46, 47). In our real-world retrospective cohort, however, PD-L1 testing was unavailable for most patients and the number of cases with documented PD-L1 scores was insufficient to support robust PD-L1 stratified analyses or treatment-by-biomarker interaction testing. This was largely attributable to three practical factors: (i) many diagnoses were established by endoscopic biopsy at other hospitals, and the original tissue blocks were unavailable for PD-L1 testing at our institution; (ii) in some cases, residual biopsy material was insufficient for additional immunohistochemistry; and (iii) routine PD-L1 assessment was not uniformly implemented. Accordingly, whether PD-L1 status modifies the benefit of neoadjuvant immunochemotherapy could not be reliably assessed here, highlighting the need for prospective studies with standardized biomarker testing and predefined treatment-selection criteria. Beyond biomarker feasibility, treatment tolerability was another practical prerequisite for the adoption of nICT.

Treatment-related adverse events (AE) during neoadjuvant therapy are summarized in **Supplementary Table 7**. The overall AE grade distribution was comparable between the nCT and nICT groups, and grade ≥3 events were not increased with nICT. Immune-related adverse events were observed only in the nICT group and were infrequent (predominantly rash; rare pneumonitis/hepatitis). These observation was in line with randomized evidence indicating acceptable tolerability of neoadjuvant PD-1 inhibitor plus chemotherapy in resectable ESCC (48).

Several additional limitations should be mentioned (1): As a single-center retrospective cohort study, selection bias and information bias were inevitable (2); Different specific drugs within the neoadjuvant categories, despite sharing similar mechanisms, might have potential inter-drug differences affecting the outcomes (3); The extent of lymphadenectomy was not uniform and likely influenced station-specific metastasis rates (4); Imaging modalities like CT had inherent false positive and negative in assessing lymph node metastasis, potentially affecting accuracy in recurrence evaluation (5); Unclear anatomical positioning during surgery leading to inaccurate lymph node station assignment and no lymph nodes were found by pathological examination after lymphadenectomy at a certain station (6); Poor compliance with postoperative follow-up in some patients resulted in untimely diagnosis and assessment.

In conclusion, compared to nCT alone, nICT provided evidence-based support for the individualized comprehensive treatment of locally advanced TESCC by enhancing the depth of pathological response, optimizing the control of lymph node metastasis, and reducing the risk of postoperative recurrence/metastasis.

## Data Availability

The raw data supporting the conclusions of this article will be made available by the authors, without undue reservation.
