# Supplementary material for: Analyses on patterns of lymph node metastasis and its impact on prognosis in thoracic esophageal squamous cell carcinoma treated with neoadjuvant immunochemotherapy versus chemotherapy alone: a single-center, retrospective cohort study
Source: Front Immunol. 2026 Mar 4;17:1762746. doi: 10.3389/fimmu.2026.1762746 (PMC12996041; doi:10.3389/fimmu.2026.1762746)
Supplement: Supplementary file 1 [file Table1.docx]

Supplementary Material

# Supplementary Figure


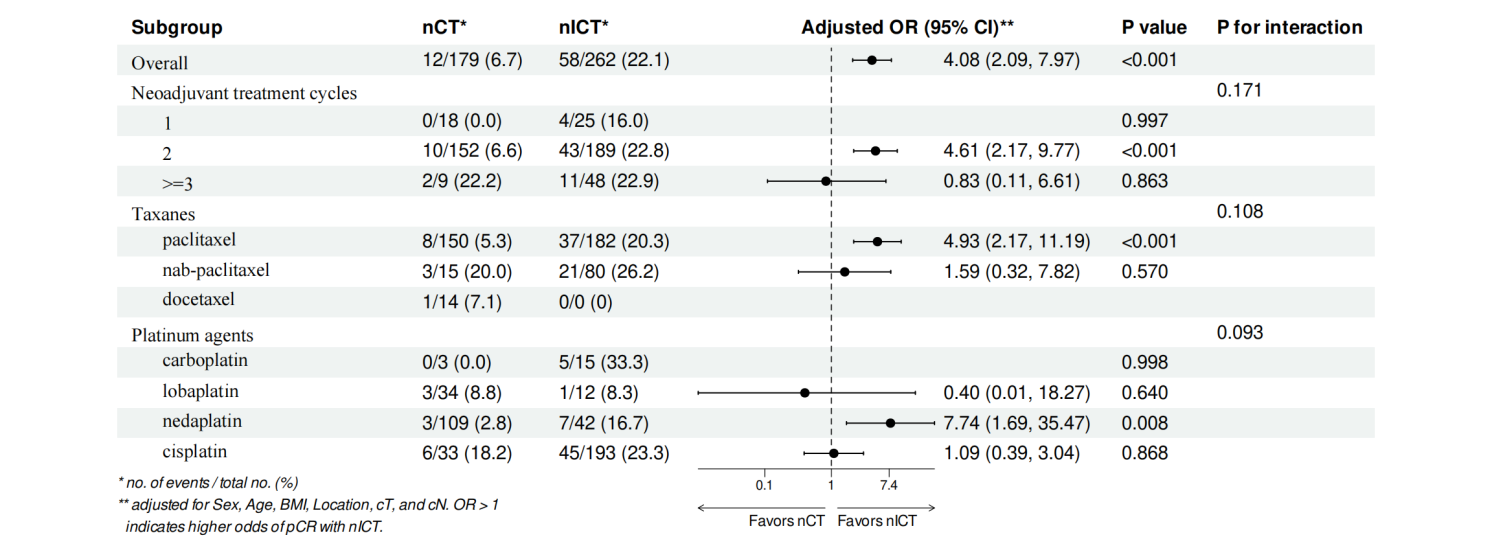


# Supplementary figure 1. Forest plot of prespecified subgroup analyses for pCR. Adjusted odds ratios (ORs) and 95% confidence intervals (CIs) for pCR comparing nICT with nCT are shown across subgroups defined by neoadjuvant treatment cycles (1, 2, ≥3), taxane backbone (paclitaxel, nab-paclitaxel, docetaxel), and platinum agent (carboplatin, lobaplatin, nedaplatin, cisplatin). ORs were estimated using multivariable logistic regression adjusted for sex, age, BMI, tumor location, clinical T stage (cT), and clinical N stage (cN). P values for interaction were derived from treatment-by-subgroup interaction terms. Subgroups with sparse data should be interpreted cautiously; estimates may be not estimable when no patients/events were present.

# Supplementary Tables (**Supplementary table 1-7**)

**Supplementary table 1. Lymphadenectomy fields and Japanese lymph node station map used in this study.**

| **Field** | **Station level** | **Station name** | **Two-field** | **Three-field** |
| --- | --- | --- | --- | --- |
| **Cervical** | 101 | Cervical paraesophageal LN | No | Yes |
| **Cervical (optional*)** | 102 | Deep cervical LN | No | Yes (if recorded) |
| **Cervical (optional*)** | 103 | Peripharyngeal / cervical peri-laryngeal LN | No | Yes (if recorded) |
| **Cervical** | 104 | Supraclavicular LN | No | Yes |
| **Thoracic** | 105 | Upper thoracic paraesophageal LN | Yes | Yes |
| **Thoracic** | 106rec (L/R) | Recurrent laryngeal nerve LN | Yes | Yes |
| **Thoracic** | 106pre | Pretracheal / paratracheal LN | Yes | Yes |
| **Thoracic** | 106tb (L/R) | Tracheobronchial LN | Yes | Yes |
| **Thoracic** | 107 | Subcarinal LN | Yes | Yes |
| **Thoracic** | 108 | Middle thoracic paraesophageal LN | Yes | Yes |
| **Thoracic** | 109 | Main bronchus LN | Yes | Yes |
| **Thoracic** | 110 | Lower thoracic paraesophageal LN | Yes | Yes |
| **Thoracic** | 111 | Supradiaphragmatic LN | Yes | Yes |
| **Thoracic** | 112 | Posterior mediastinal LN (±subgroups) | Yes | Yes |
| **Abdominal** | 1 | Right paracardial perigastric LN | Yes | Yes |
| **Abdominal** | 2 | Left paracardial perigastric LN | Yes | Yes |
| **Abdominal** | 3 | Lesser curvature perigastric LN | Yes | Yes |
| **Abdominal** | 7 | Along left gastric artery LN | Yes | Yes |
| **Abdominal** | 9 | Along celiac artery LN | Yes | Yes |
| **Abdominal** | 19 | Infradiaphragmatic LN | Yes | Yes |
| **Abdominal** | 20 | Paraesophageal LN at the esophageal hiatus | Yes | Yes |
| **Abdominal (optional**)** | 8a | Common hepatic artery LN | Optional | Optional |
| **Abdominal (optional**)** | 11p | Proximal splenic artery LN | Optional | Optional |
| Footnotes：* Cervical stations 102/103 were included only if routinely separated and recorded at our institution; otherwise, three-field cervical dissection included at minimum stations 101 and 104. ** Abdominal vascular stations (e.g., 8a/11p) were dissected when clinically indicated according to intraoperative findings or preoperative assessment. Detailed anatomical definitions follow the Japanese Classification of Esophageal Cancer (JES, 12th edition) for cervical/thoracic stations. | | | | |

**Supplementary table 2. Detailed neoadjuvant regimens and PD-1 inhibitors used in the cohort.**

| **Characteristic** | **Group** | |
| --- | --- | --- |
|  | **nCT^1^** | **nICT^2^** |
|  | **(N = 179)** | **(N = 262)** |
| **Taxane type, n (%)** |  |  |
| paclitaxel | 150 (83.8%) | 182 (69.5%) |
| nab-paclitaxel | 15 (8.4%) | 80 (30.5%) |
| docetaxel | 14 (7.8%) | 0 (0.0%) |
| **Platinum type, n (%)** |  |  |
| carboplatin | 3 (1.7%) | 15 (5.7%) |
| nedaplatin | 109 (60.9%) | 42 (16.0%) |
| lobaplatin | 34 (19.0%) | 12 (4.6%) |
| cisplatin | 33 (18.4%) | 193 (73.7%) |
| **PD-1 agent, n (%)** |  |  |
| sintilimab | NA | 26 (9.9%) |
| camrelizumab | NA | 41 (15.6%) |
| pembrolizumab | NA | 38 (14.5%) |
| serplulimab | NA | 20 (7.6%) |
| tislelizumab | NA | 123 (46.9%) |
| toripalimab | NA | 14 (5.3%) |
| **neoadjuvant therapy cycles, n (%)** |  |  |
| 1 | 18 (10.1%) | 25 (9.5%) |
| 2 | 152 (84.9%) | 189 (72.1%) |
| ˃=3 | 9 (5.0%) | 48 (18.3%) |
| ^1^nICT: neoadjuvant immunochemotherapy; ^2^nCT: neoadjuvant chemotherapy | | |

**Supplementary table 3. Comparison of the number of lymph node dissections and metastases.**

| **Characteristic** | **Dissected lymph nodes** | | **p-value^1^** | **Metastatic lymph nodes** | | **p-value^1^** |
| --- | --- | --- | --- | --- | --- | --- |
|  | nCT | nICT |  | nCT | nICT |  |
|  | **N = 179** | **N = 262** |  | **N = 179** | **N = 262** |  |
| **Number of LN,** Median (Q1, Q3) | 26.0 (17.0, 35.0) | 36.0 (26.0, 47.0) | <0.001 | 0.0 (0.0, 2.0) | 0.0 (0.0, 1.0) | 0.083 |
| **LN_101R,** Median (Q1, Q3) | 1.0 (1.0, 3.0) | 1.0 (1.0, 2.0) | 0.663 | 1.0 (1.0, 1.0) | 1.0 (1.0, 2.0) | 0.402 |
| **LN_101L,** Median (Q1, Q3) | 1.0 (1.0, 2.0) | 2.0 (1.0, 2.0) | 0.114 | 1.0 (1.0, 1.0) | 1.0 (1.0, 2.0) | 0.265 |
| **LN_104R,** Median (Q1, Q3) | 4.0 (2.0, 7.0) | 6.0 (2.0, 11.0) | 0.263 | 1.5 (1.0, 2.5) | 1.0 (1.0, 2.0) | 0.574 |
| **LN_104L,** Median (Q1, Q3) | 6.0 (3.0, 9.0) | 9.0 (4.0, 14.0) | 0.066 | 1.0 (1.0, 2.0) | 1.0 (1.0, 1.5) | 0.334 |
| **LN_105,** Median (Q1, Q3) | 1.0 (1.0, 2.0) | 2.0 (1.0, 3.0) | 0.172 | - | - | - |
| **LN_106recR,** Median (Q1, Q3) | 1.0 (1.0, 2.0) | 2.00 (1.0, 4.0) | <0.001 | 1.0 (1.0, 1.0) | 1.0 (1.0, 1.0) | 0.882 |
| **LN_106recL,** Median (Q1, Q3) | 2.0 (1.0, 3.0) | 3.0 (1.0, 4.0) | <0.001 | - | - | - |
| **LN_106tbL,** Median (Q1, Q3) | 2.0 (1.0, 4.0) | 2.0 (1.0, 5.0) | 0.403 | - | - | - |
| **LN_107,** Median (Q1, Q3) | 2.0 (1.0, 4.0) | 4.0 (2.0, 6.5) | <0.001 | 1.0 (1.0, 2.0) | 1.0 (1.0, 1.0) | 0.121 |
| **LN_108,** Median (Q1, Q3) | 1.0 (1.0, 2.5) | 2.0 (1.0, 3.0) | 0.208 | 1.0 (1.0, 1.0) | 1.0 (1.0, 2.0) | 0.530 |
| **LN_109R,** Median (Q1, Q3) | 1.0 (1.0, 2.0) | 1.5 (1.0, 2.0) | 0.002 | - | - | - |
| **LN_109L,** Median (Q1, Q3) | 1.0 (1.0, 2.0) | 2.0 (1.0, 2.0) | 0.006 | - | - | - |
| **LN_110,** Median (Q1, Q3) | 1.0 (1.0, 2.0) | 2.0 (1.0, 3.0) | 0.082 | 1.0 (1.0, 3.0) | 1.0 (1.0, 1.0) | 0.220 |
| **LN_111,** Median (Q1, Q3) | 1.0 (1.0, 2.0) | 1.0 (1.0, 3.0) | 0.047 | - | - | - |
| **LN_112,** Median (Q1, Q3) | 1.0 (1.0, 2.0) | 1.0 (1.0, 2.0) | 0.249 | 1.0 (1.0, 1.0) | 1.0 (1.0, 1.0) | 0.126 |
| **LN_1,** Median (Q1, Q3) | 3.0 (1.0, 6.0) | 7.0 (4.0, 13.0) | <0.001 | 1.0 (1.0, 2.0) | 1.0 (1.0, 2.0) | 0.844 |
| **LN_2,** Median (Q1, Q3) | 2.0 (1.0, 5.0) | 2.0 (1.0, 4.0) | 0.751 | 1.0 (1.0, 2.0) | 1.0 (1.0, 1.0) | 0.299 |
| **LN_3,** Median (Q1, Q3) | 4.0 (2.0, 6.0) | 3.0 (2.0, 6.0) | 0.420 | - | - | - |
| **LN_7,** Median (Q1, Q3) | 3.0 (1.0, 4.0) | 3.0 (2.0, 6.0) | 0.002 | 1.0 (1.0, 1.0) | 1.0 (1.0, 1.0) | 0.987 |
| **LN_8,** Median (Q1, Q3) | 2.0 (1.00, 3.0) | 2.0 (1.0, 4.0) | 0.212 | 1.0 (1.0, 3.0) | 1.5 (1.0, 3.5) | 0.845 |
| ^1^Wilcoxon rank sum test; LN, lymph node. | | | | | | |

**Supplementary table 4. Comparison of lymph node ratio (LNR/LNMR1) before and after propensity score matching (PSM).**

| **Characteristic** | **Unmatched** | | | **Matched** | | |
| --- | --- | --- | --- | --- | --- | --- |
|  | **nCT  N = 179** | **nICT  N = 262** | **p-value** | **nCT  N = 179** | **nICT  N = 172** | **p-value** |
| **LNMR1_101R**, Median (Q1, Q3) | 0.032 (0.026, 0.059) | 0.022 (0.014, 0.049) | 0.166^1^ | 0.032 (0.026, 0.059) | 0.054 (0.024, 0.063) | 0.510^1^ |
| **LNMR1_101L**, Median (Q1, Q3) | 0.040 (0.033, 0.050) | 0.035 (0.018, 0.049) | 0.625^1^ | 0.040 (0.033, 0.050) | 0.038 (0.031, 0.049) | 0.951^1^ |
| **LNMR1_104R**, Median (Q1, Q3) | 0.034 (0.028, 0.075) | 0.027 (0.023, 0.038) | 0.225^1^ | 0.034 (0.028, 0.075) | 0.031 (0.024, 0.040) | 0.521^1^ |
| **LNMR1_104L**, Median (Q1, Q3) | 0.061 (0.031, 0.071) | 0.026 (0.020, 0.033) | 0.048^1^ | 0.061 (0.031, 0.071) | 0.026 (0.026, 0.026) | 0.109^1^ |
| **LNMR1_105**, Median (Q1, Q3) | 0.026 (0.015, 0.034) | 0.024 (0.018, 0.031) | 0.957^1^ | 0.026 (0.015, 0.034) | 0.030 (0.018, 0.063) | 0.431^1^ |
| **LNMR1_106recR**, Median (Q1, Q3) | 0.039 (0.030, 0.048) | 0.021 (0.014, 0.032) | <0.001^1^ | 0.039 (0.030, 0.048) | 0.031 (0.023, 0.043) | 0.178^1^ |
| **LNMR1_106recL**, Median (Q1, Q3) | 0.037 (0.021, 0.061) | 0.022 (0.017, 0.032) | 0.092^1^ | 0.037 (0.021, 0.061) | 0.028 (0.021, 0.037) | 0.310^1^ |
| **LNMR1_106tbL**, Median (Q1, Q3) | 0.036 (0.034, 0.037) | 0.017 (0.017, 0.017) | 0.333^2^ | 0.0357 (0.0345, 0.0370) | NA (NA, NA) | - |
| **LNMR1_107**, Median (Q1, Q3) | 0.045 (0.030, 0.051) | 0.022 (0.019, 0.032) | <0.001^1^ | 0.045 (0.030, 0.051) | 0.026 (0.021, 0.050) | 0.122^1^ |
| **LNMR1_108**, Median (Q1, Q3) | 0.029 (0.024, 0.056) | 0.028 (0.020, 0.037) | 0.426^1^ | 0.029 (0.024, 0.056) | 0.030 (0.025, 0.048) | 0.845^1^ |
| **LNMR1_109R**, Median (Q1, Q3) | 0.031 (0.028, 0.034) | 0.013 (0.013, 0.013) | 0.333^2^ | NA (NA, NA) | NA (NA, NA) | - |
| **LNMR1_109L**, Median (Q1, Q3) | 0.048 (0.030, 0.071) | 0.021 (0.017, 0.026) | 0.057^2^ | 0.048 (0.030, 0.071) | 0.025 (0.020, 0.029) | 0.200^1^ |
| **LNMR1_110**, Median (Q1, Q3) | 0.045 (0.025, 0.085) | 0.022 (0.011, 0.032) | 0.032^1^ | 0.05 (0.03, 0.09) | 0.03 (0.02, 0.03) | 0.239^1^ |
| **LNMR1_111**, Median (Q1, Q3) | 0.031 (0.012, 0.100) | 0.035 (0.015, 0.063) | 0.857^2^ | 0.031 (0.012, 0.100) | 0.063 (0.050, 0.077) | 0.800^1^ |
| **LNMR1_112**, Median (Q1, Q3) | 0.042 (0.038, 0.048) | 0.028 (0.024, 0.045) | 0.238^1^ | 0.042 (0.038, 0.048) | 0.042 (0.026, 0.077) | 0.683^1^ |
| **LNMR1_1**, Median (Q1, Q3) | 0.050 (0.036, 0.064) | 0.030 (0.023, 0.050) | 0.025^1^ | 0.05 (0.04, 0.06) | 0.04 (0.03, 0.06) | 0.213^1^ |
| **LNMR1_2**, Median (Q1, Q3) | 0.053 (0.032, 0.061) | 0.036 (0.025, 0.043) | 0.012^1^ | 0.053 (0.032, 0.061) | 0.036 (0.026, 0.043) | 0.027^1^ |
| **LNMR1_3**, Median (Q1, Q3) | 0.077 (0.074, 0.080) | 0.023 (0.016, 0.028) | 0.133^2^ | 0.077 (0.074, 0.080) | 0.024 (0.021, 0.032) | 0.200^1^ |
| **LNMR1_7**, Median (Q1, Q3) | 0.039 (0.031, 0.065) | 0.035 (0.025, 0.048) | 0.240^1^ | 0.039 (0.031, 0.065) | 0.037 (0.030, 0.051) | 0.682^1^ |
| **LNMR1_8**, Median (Q1, Q3) | 0.063 (0.029, 0.097) | 0.031 (0.018, 0.047) | 0.229^2^ | 0.063 (0.029, 0.097) | NA (NA, NA) | - |
| **LNMR1_11**, Median (Q1, Q3) | 0.059 (0.037, 0.069) | NA (NA, NA) |  | 0.059 (0.037, 0.069) | NA (NA, NA) | - |
| ^1^Wilcoxon rank sum test; ^2^Wilcoxon rank sum exact test; ^3^Pearson's Chi-squared test; LNMR1, lymph node ratio. | | | | | | |

**Supplementary table 5. Comparison of lymph node metastasis rate 2 and 3 (LNMR2 and LNMR3) before propensity score matching (PSM).**

| **LN_Station** | **Group** | | **p-value** | **Group** | | **p-value** |
| --- | --- | --- | --- | --- | --- | --- |
|  | **nCT  N = 179** | **nICT  N = 262** |  | **nCT  N = 179** | **nICT  N = 262** |  |
|  | **LNMR2** | |  | **LNMR3** | |  |
| **LN_101R**, n (%) |  |  | 0.409^1^ |  |  | 0.884^1^ |
| 0 | 166 (92.7%) | 248 (94.7%) |  | 69 (84.1%) | 79 (84.9%) |  |
| 1 | 13 (7.3%) | 14 (5.3%) |  | 13 (15.9%) | 14 (15.1%) |  |
| **LN_101L**, n (%) |  |  | 0.069^1^ |  |  | 0.248^1^ |
| 0 | 169 (94.4%) | 256 (97.7%) |  | 46 (82.1%) | 52 (89.7%) |  |
| 1 | 10 (5.6%) | 6 (2.3%) |  | 10 (17.9%) | 6 (10.3%) |  |
| **LN_104R**, n (%) |  |  | 0.196^1^ |  |  | 0.411^1^ |
| 0 | 175 (97.8%) | 250 (95.4%) |  | 24 (85.7%) | 43 (78.2%) |  |
| 1 | 4 (2.2%) | 12 (4.6%) |  | 4 (14.3%) | 12 (21.8%) |  |
| **LN_104L**, n (%) |  |  | 0.290^1^ |  |  | 0.126^1^ |
| 0 | 170 (95.0%) | 254 (96.9%) |  | 28 (75.7%) | 56 (87.5%) |  |
| 1 | 9 (5.0%) | 8 (3.1%) |  | 9 (24.3%) | 8 (12.5%) |  |
| **LN_105**, n (%) |  |  | 0.798^1^ |  |  | 0.925^1^ |
| 0 | 173 (96.6%) | 252 (96.2%) |  | 61 (91.0%) | 107 (91.5%) |  |
| 1 | 6 (3.4%) | 10 (3.8%) |  | 6 (9.0%) | 10 (8.5%) |  |
| **LN_106recR**, n (%) |  |  | 0.924^1^ |  |  | 0.883^1^ |
| 0 | 157 (87.7%) | 229 (87.4%) |  | 102 (82.3%) | 160 (82.9%) |  |
| 1 | 22 (12.3%) | 33 (12.6%) |  | 22 (17.7%) | 33 (17.1%) |  |
| **LN_106recL**, n (%) |  |  | 0.241^1^ |  |  | 0.409^1^ |
| 0 | 172 (96.1%) | 245 (93.5%) |  | 89 (92.7%) | 147 (89.6%) |  |
| 1 | 7 (3.9%) | 17 (6.5%) |  | 7 (7.3%) | 17 (10.4%) |  |
| **LN_106tbL**, n (%) |  |  | 0.043^2^ |  |  | 0.200^2^ |
| 0 | 174 (97.2%) | 261 (99.6%) |  | 34 (87.2%) | 38 (97.4%) |  |
| 1 | 5 (2.8%) | 1 (0.4%) |  | 5 (12.8%) | 1 (2.6%) |  |
| **LN_107**, n (%) |  |  | 0.569^1^ |  |  | 0.456^1^ |
| 0 | 156 (87.2%) | 233 (88.9%) |  | 139 (85.8%) | 219 (88.3%) |  |
| 1 | 23 (12.8%) | 29 (11.1%) |  | 23 (14.2%) | 29 (11.7%) |  |
| **LN_108**, n (%) |  |  | 0.554^1^ |  |  | 0.487^1^ |
| 0 | 164 (91.6%) | 244 (93.1%) |  | 105 (87.5%) | 163 (90.1%) |  |
| 1 | 15 (8.4%) | 18 (6.9%) |  | 15 (12.5%) | 18 (9.9%) |  |
| **LN_109R**, n (%) |  |  | >0.999^2^ |  |  | >0.999^2^ |
| 0 | 177 (98.9%) | 260 (99.2%) |  | 62 (96.9%) | 70 (97.2%) |  |
| 1 | 2 (1.1%) | 2 (0.8%) |  | 2 (3.1%) | 2 (2.8%) |  |
| **LN_109L**, n (%) |  |  | >0.999^2^ |  |  | >0.999^2^ |
| 0 | 176 (98.3%) | 258 (98.5%) |  | 76 (96.2%) | 117 (96.7%) |  |
| 1 | 3 (1.7%) | 4 (1.5%) |  | 3 (3.8%) | 4 (3.3%) |  |
| **LN_110**, n (%) |  |  | 0.116^1^ |  |  | 0.064^1^ |
| 0 | 168 (93.9%) | 254 (96.9%) |  | 86 (88.7%) | 150 (94.9%) |  |
| 1 | 11 (6.1%) | 8 (3.1%) |  | 11 (11.3%) | 8 (5.1%) |  |
| **LN_111**, n (%) |  |  | >0.999^2^ |  |  | 0.692^2^ |
| 0 | 176 (98.3%) | 258 (98.5%) |  | 68 (95.8%) | 134 (97.1%) |  |
| 1 | 3 (1.7%) | 4 (1.5%) |  | 3 (4.2%) | 4 (2.9%) |  |
| **LN_112**, n (%) |  |  | 0.179^1^ |  |  | 0.490^1^ |
| 0 | 168 (93.9%) | 253 (96.6%) |  | 56 (83.6%) | 64 (87.7%) |  |
| 1 | 11 (6.1%) | 9 (3.4%) |  | 11 (16.4%) | 9 (12.3%) |  |
| **LN_1**, n (%) |  |  | 0.712^1^ |  |  | 0.769^1^ |
| 0 | 160 (89.4%) | 237 (90.5%) |  | 141 (88.1%) | 204 (89.1%) |  |
| 1 | 19 (10.6%) | 25 (9.5%) |  | 19 (11.9%) | 25 (10.9%) |  |
| **LN_2**, n (%) |  |  | 0.108^1^ |  |  | 0.442^1^ |
| 0 | 158 (88.3%) | 243 (92.7%) |  | 153 (87.9%) | 179 (90.4%) |  |
| 1 | 21 (11.7%) | 19 (7.3%) |  | 21 (12.1%) | 19 (9.6%) |  |
| **LN_3**, n (%) |  |  | >0.999^2^ |  |  | 0.692^2^ |
| 0 | 177 (98.9%) | 258 (98.5%) |  | 70 (97.2%) | 85 (95.5%) |  |
| 1 | 2 (1.1%) | 4 (1.5%) |  | 2 (2.8%) | 4 (4.5%) |  |
| **LN_7**, n (%) |  |  | 0.020^1^ |  |  | 0.012^1^ |
| 0 | 153 (85.5%) | 242 (92.4%) |  | 129 (83.2%) | 218 (91.6%) |  |
| 1 | 26 (14.5%) | 20 (7.6%) |  | 26 (16.8%) | 20 (8.4%) |  |
| **LN_8**, n (%) |  |  | >0.999^2^ |  |  | 0.373^2^ |
| 0 | 176 (98.3%) | 258 (98.5%) |  | 35 (92.1%) | 106 (96.4%) |  |
| 1 | 3 (1.7%) | 4 (1.5%) |  | 3 (7.9%) | 4 (3.6%) |  |
| **LN_9**, n (%) |  |  | 0.164^2^ |  |  | 0.109^2^ |
| 0 | 177 (98.9%) | 262 (100.0%) |  | 2 (50.0%) | 7 (100.0%) |  |
| 1 | 2 (1.1%) | 0 (0.0%) |  | 2 (50.0%) | 0 (0.0%) |  |
| **LN_11**, n (%) |  |  | 0.066^2^ |  |  | 0.200^2^ |
| 0 | 176 (98.3%) | 262 (100.0%) |  | 5 (62.5%) | 7 (100.0%) |  |
| 1 | 3 (1.7%) | 0 (0.0%) |  | 3 (37.5%) | 0 (0.0%) |  |
| ^1^Pearson's Chi-squared test; ^2^Fisher's exact test; “0” indicates the number of patients without lymph node metastasis and “1” indicates the number of patients with lymph node metastasis; LNMR2/LNMR3, lymph node metastasis rate 2 or 3. | | | | | | |

**Supplementary table 6. Comparison of lymph node metastasis rate 2 and 3 (LNMR2 and LNMR3) after propensity score matching (PSM).**

| **LN_Station** | **Unmatched** | | **p-value** | **Matched** | | **p-value** |
| --- | --- | --- | --- | --- | --- | --- |
|  | **nCT  N = 179** | **nICT  N = 262** |  | **nCT  N = 179** | **nICT  N = 262** |  |
|  | **LNMR2** | |  | **LNMR3** | |  |
| **LN_101R**, n (%) |  |  | 0.099^1^ |  |  | 0.634^1^ |
| 0 | 166 (92.7%) | 173 (96.6%) |  | 69 (84.1%) | 41 (87.2%) |  |
| 1 | 13 (7.3%) | 6 (3.4%) |  | 13 (15.9%) | 6 (12.8%) |  |
| **LN_101L**, n (%) |  |  | 0.187^1^ |  |  | 0.742^1^ |
| 0 | 169 (94.4%) | 174 (97.2%) |  | 46 (82.1%) | 28 (84.8%) |  |
| 1 | 10 (5.6%) | 5 (2.8%) |  | 10 (17.9%) | 5 (15.2%) |  |
| **LN_104R**, n (%) |  |  | 0.521^1^ |  |  | 0.734^2^ |
| 0 | 175 (97.8%) | 173 (96.6%) |  | 24 (85.7%) | 25 (80.6%) |  |
| 1 | 4 (2.2%) | 6 (3.4%) |  | 4 (14.3%) | 6 (19.4%) |  |
| **LN_104L**, n (%) |  |  | 0.275^1^ |  |  | 0.443^1^ |
| 0 | 170 (95.0%) | 174 (97.2%) |  | 28 (75.7%) | 25 (83.3%) |  |
| 1 | 9 (5.0%) | 5 (2.8%) |  | 9 (24.3%) | 5 (16.7%) |  |
| **LN_105**, n (%) |  |  | 0.778^1^ |  |  | 0.835^1^ |
| 0 | 173 (96.6%) | 172 (96.1%) |  | 61 (91.0%) | 63 (90.0%) |  |
| 1 | 6 (3.4%) | 7 (3.9%) |  | 6 (9.0%) | 7 (10.0%) |  |
| **LN_106recR**, n (%) |  |  | 0.396^1^ |  |  | 0.328^1^ |
| 0 | 157 (87.7%) | 162 (90.5%) |  | 102 (82.3%) | 111 (86.7%) |  |
| 1 | 22 (12.3%) | 17 (9.5%) |  | 22 (17.7%) | 17 (13.3%) |  |
| **LN_106recL**, n (%) |  |  | 0.238^1^ |  |  | 0.327^1^ |
| 0 | 172 (96.1%) | 167 (93.3%) |  | 89 (92.7%) | 94 (88.7%) |  |
| 1 | 7 (3.9%) | 12 (6.7%) |  | 7 (7.3%) | 12 (11.3%) |  |
| **LN_106tbL**, n (%) |  |  | 0.061^2^ |  |  | 0.161^2^ |
| 0 | 174 (97.2%) | 179 (100.0%) |  | 34 (87.2%) | 19 (100.0%) |  |
| 1 | 5 (2.8%) | 0 (0.0%) |  | 5 (12.8%) | 0 (0.0%) |  |
| **LN_107**, n (%) |  |  | 0.314^1^ |  |  | 0.248^1^ |
| 0 | 156 (87.2%) | 162 (90.5%) |  | 139 (85.8%) | 152 (89.9%) |  |
| 1 | 23 (12.8%) | 17 (9.5%) |  | 23 (14.2%) | 17 (10.1%) |  |
| **LN_108**, n (%) |  |  | 0.548^1^ |  |  | 0.540^1^ |
| 0 | 164 (91.6%) | 167 (93.3%) |  | 105 (87.5%) | 108 (90.0%) |  |
| 1 | 15 (8.4%) | 12 (6.7%) |  | 15 (12.5%) | 12 (10.0%) |  |
| **LN_109R**, n (%) |  |  | 0.499^2^ |  |  | 0.528^2^ |
| 0 | 177 (98.9%) | 179 (100.0%) |  | 62 (96.9%) | 38 (100.0%) |  |
| 1 | 2 (1.1%) | 0 (0.0%) |  | 2 (3.1%) | 0 (0.0%) |  |
| **LN_109L**, n (%) |  |  | >0.999^2^ |  |  | >0.999^2^ |
| 0 | 176 (98.3%) | 177 (98.9%) |  | 76 (96.2%) | 74 (97.4%) |  |
| 1 | 3 (1.7%) | 2 (1.1%) |  | 3 (3.8%) | 2 (2.6%) |  |
| **LN_110**, n (%) |  |  | 0.065^1^ |  |  | 0.057^1^ |
| 0 | 168 (93.9%) | 175 (97.8%) |  | 86 (88.7%) | 94 (95.9%) |  |
| 1 | 11 (6.1%) | 4 (2.2%) |  | 11 (11.3%) | 4 (4.1%) |  |
| **LN_111**, n (%) |  |  | >0.999^2^ |  |  | 0.658^2^ |
| 0 | 176 (98.3%) | 177 (98.9%) |  | 68 (95.8%) | 85 (97.7%) |  |
| 1 | 3 (1.7%) | 2 (1.1%) |  | 3 (4.2%) | 2 (2.3%) |  |
| **LN_112**, n (%) |  |  | 0.333^1^ |  |  | 0.567^1^ |
| 0 | 168 (93.9%) | 172 (96.1%) |  | 56 (83.6%) | 48 (87.3%) |  |
| 1 | 11 (6.1%) | 7 (3.9%) |  | 11 (16.4%) | 7 (12.7%) |  |
| **LN_1**, n (%) |  |  | >0.999^1^ |  |  | 0.883^1^ |
| 0 | 160 (89.4%) | 160 (89.4%) |  | 141 (88.1%) | 134 (87.6%) |  |
| 1 | 19 (10.6%) | 19 (10.6%) |  | 19 (11.9%) | 19 (12.4%) |  |
| **LN_2**, n (%) |  |  | 0.385^1^ |  |  | 0.989^1^ |
| 0 | 158 (88.3%) | 163 (91.1%) |  | 153 (87.9%) | 116 (87.9%) |  |
| 1 | 21 (11.7%) | 16 (8.9%) |  | 21 (12.1%) | 16 (12.1%) |  |
| **LN_3**, n (%) |  |  | >0.999^2^ |  |  | 0.371^2^ |
| 0 | 177 (98.9%) | 176 (98.3%) |  | 70 (97.2%) | 42 (93.3%) |  |
| 1 | 2 (1.1%) | 3 (1.7%) |  | 2 (2.8%) | 3 (6.7%) |  |
| **LN_7**, n (%) |  |  | 0.143^1^ |  |  | 0.103^1^ |
| 0 | 153 (85.5%) | 162 (90.5%) |  | 129 (83.2%) | 145 (89.5%) |  |
| 1 | 26 (14.5%) | 17 (9.5%) |  | 26 (16.8%) | 17 (10.5%) |  |
| **LN_8**, n (%) |  |  | 0.248^2^ |  |  | 0.057^2^ |
| 0 | 176 (98.3%) | 179 (100.0%) |  | 35 (92.1%) | 59 (100.0%) |  |
| 1 | 3 (1.7%) | 0 (0.0%) |  | 3 (7.9%) | 0 (0.0%) |  |
| **LN_9**, n (%) |  |  | 0.499^2^ |  |  | 0.429^2^ |
| 0 | 177 (98.9%) | 179 (100.0%) |  | 2 (50.0%) | 3 (100.0%) |  |
| 1 | 2 (1.1%) | 0 (0.0%) |  | 2 (50.0%) | 0 (0.0%) |  |
| **LN_11**, n (%) |  |  | 0.248^2^ |  |  | 0.491^2^ |
| 0 | 176 (98.3%) | 179 (100.0%) |  | 5 (62.5%) | 4 (100.0%) |  |
| 1 | 3 (1.7%) | 0 (0.0%) |  | 3 (37.5%) | 0 (0.0%) |  |
| ^1^Pearson's Chi-squared test; ^2^Fisher's exact test; “0” indicates the number of patients without lymph node metastasis and “1” indicates the number of patients with lymph node metastasis; LNMR2/LNMR3, lymph node metastasis rate 2 or 3. | | | | | | |

### **Supplementary table 7. Treatment-related adverse events during neoadjuvant therapy.**

| **Category** | **Adverse event** | **nCT (N=179), n (%)** | **nICT (N=262), n (%)** | **P value^1^** |
| --- | --- | --- | --- | --- |
| **Overall AE severity (grade)** | **Overall distribution** | — | — | 0.132 |
|  | None | 42 (23.5%) | 84 (32.3%) | — |
|  | Grade 1–2 | 112 (62.6%) | 144 (55.4%) | — |
|  | Grade ≥3 | 25 (14.0%) | 32 (12.3%) | — |
| **Preoperative immune-related AEs (irAEs)** | Rash | NA | 33 (12.6%) | — |
|  | Immune pneumonitis | NA | 2 (0.8%) | — |
|  | Immune-related hepatitis | NA | 2 (0.8%) | — |
|  | Immune-related colitis | NA | 1 (0.4%) | — |
|  | Immune pancreatitis | NA | 1 (0.4%) | — |
|  | Myasthenia | NA | 1 (0.4%) | — |
| **Chemotherapy-related AEs** | Leukopenia | 66 (36.9%) | 101 (38.5%) | 0.797 |
|  | Neutropenia | 60 (33.5%) | 88 (33.6%) | 1 |
|  | Hemoglobin decreased | 20 (11.2%) | 43 (16.4%) | 0.16 |
|  | Transaminase elevation | 23 (12.8%) | 30 (11.5%) | 0.768 |
|  | Gastrointestinal reactions | 54 (30.2%) | 52 (19.8%) | 0.017 |
|  | Fatigue | 15 (8.4%) | 35 (13.4%) | 0.143 |
|  | Alopecia | 19 (10.6%) | 25 (9.5%) | 0.836 |
|  | Other^2^ | 7 (3.9%) | 25 (9.5%) | - |
| **Abbreviations:** AE, adverse event; irAE, immune-related adverse event; nCT, neoadjuvant chemotherapy; nICT, neoadjuvant immunochemotherapy; NA, not applicable/not available. **Footnotes：^1^** Pearson’s chi-square test; ^2^ **Other** includes hyponatremia, hypokalemia, elevated creatinine, lower limb pain, thrombocytopenia, and allergic reactions (e.g., chest tightness, diaphoresis). | | | | |
